# Testing the event witnessing status of micro-bloggers from evidence in their micro-blogs

**DOI:** 10.1371/journal.pone.0189378

**Published:** 2017-12-12

**Authors:** Marie Truelove, Maria Vasardani, Stephan Winter

**Affiliations:** Department of Infrastructure Engineering, The University of Melbourne, Parkville, Victoria, Australia; Waseda University, JAPAN

## Abstract

This paper demonstrates a framework of processes for identifying potential witnesses of events from evidence they post to social media. The research defines original evidence models for micro-blog content sources, the relative uncertainty of different evidence types, and models for testing evidence by combination. Methods to filter and extract evidence using automated and semi-automated means are demonstrated using a Twitter case study event. Further, an implementation to test extracted evidence using Dempster Shafer Theory of Evidence are presented. The results indicate that the inclusion of evidence from micro-blog text and linked image content can increase the number of micro-bloggers identified at events, in comparison to the number of micro-bloggers identified from geotags alone. Additionally, the number of micro-bloggers that can be tested for evidence corroboration or conflict, is increased by incorporating evidence identified in their posting history.

## Introduction

Distinguishing social media posts that originate from witnesses on-the-ground (OTG) in contrast to micro-bloggers merely posting commentary from afar contributes to numerous application domains, including journalism [1] and emergency management [2]. In addition to gaining information about events, witness accounts suggest increased relevance and credibility compared to information posted from a source who is not a witness [3]. This notion is founded in disciplines that research credibility of crowdsourced information, such as Volunteered Geographic Information (VGI) or Citizen Journalism, that describe contributors with local knowledge in the proximity of time-critical events compared to domain experts [1, 4].

**Fig 1:**
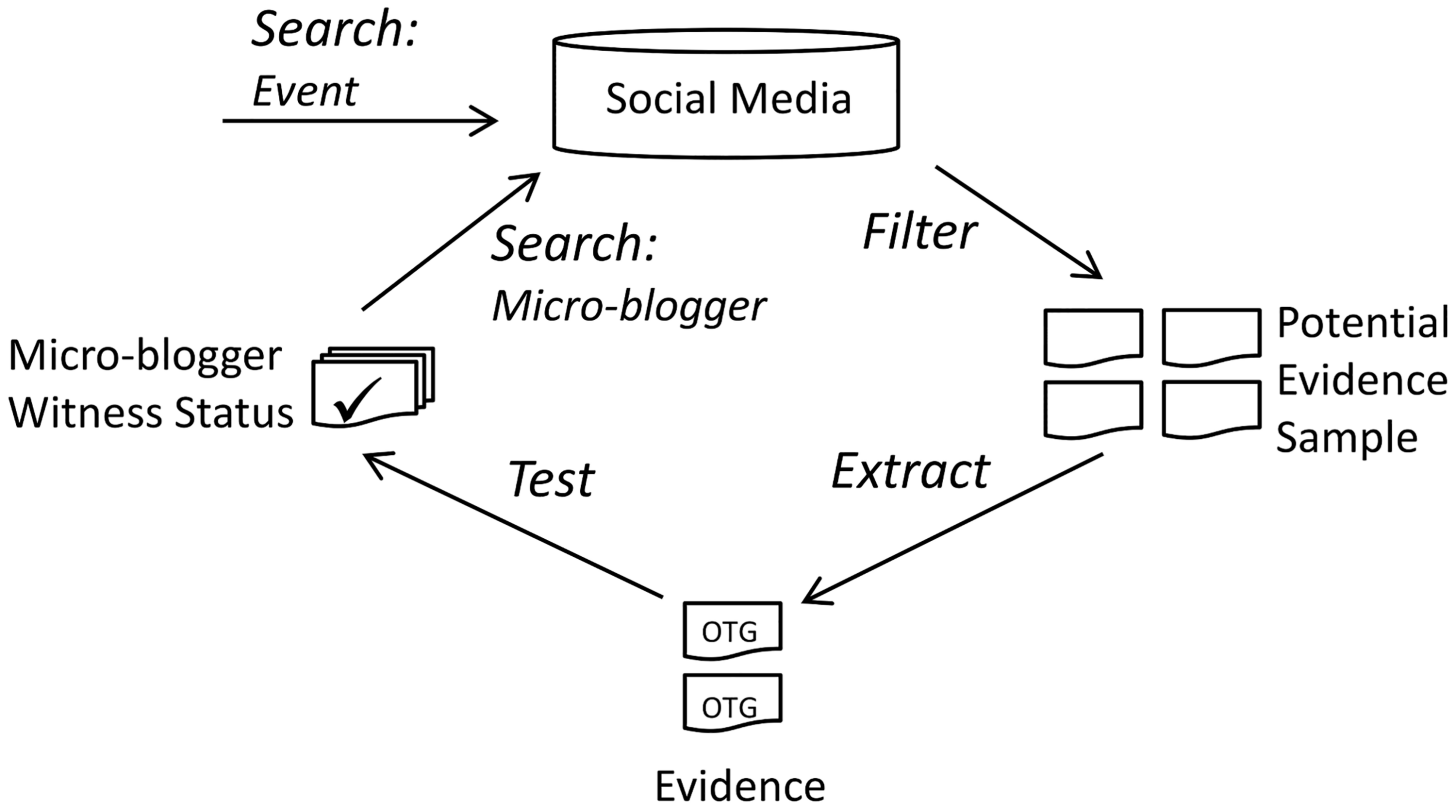
A framework of processes for identifying potential witnesses of events from evidence in micro-blogs.

Previous research has used in-depth human analysis to identify and describe characteristics that distinguish individual micro-blogs as Witness Accounts (WA) and inform a model of micro-blog categories [3, 5]. Initial consideration of WA content as evidence, and preliminary experiments to extract and test this evidence by micro-blog [6] are expanded in this study substantially. A primary contribution of this study is to demonstrate for the first time a complete framework of processes for identifying potential witnesses of events, from evidence discovered in their micro-blogs. Inspired by the judiciary system, this research has developed a framework that represents an investigator gathering evidence to support or dismiss a hypothesis of the micro-blogger’s *witnessing status*. The evidence is tested to measure its balance towards a hypothesis, and further evidence is sought about micro-bloggers of interest to improve the certainty of the results. Fig 1 labels the major processes of the framework for identifying potential witnesses, which are conceived as a cycle beginning with *search event*.

A subsequent equally important contribution of this paper is an original formal model of evidence defining evidence types and inference categories, and evidence combination test result categories, by micro-blog and micro-blogger. This model enables varying implementations for the *test* processes of the framework to be founded on the same concepts. This model supports evidence extracted from a micro-blog’s text, linked image, or geotag content, that can be classified as supporting inferences the micro-blogger is OTG or not OTG (NOTG) [6]. A Dempster Shafer Theory of Evidence (DST) implementation of the evidence model that accounts for the combination of evidence by micro-blogger and includes text and image evidence resulting from supervised classification experiments, is presented for the first time. Implementations to test the evidence must accommodate varying levels of uncertainty, which can be attributed to the content source, and the extraction process or measurement error associated with the content source. Additionally, seeking evidence of witnessing to test a micro-blogger’s status from a number of micro-blog content sources distinguishes this work in comparison to previous research e.g. [7–10].

A secondary contribution of this paper is exploring the potential of the *search micro-blogger* processes. The search micro-blogger processes are distinguished from the search event processes that provide event relevant micro-blogs from hashtag or keyword searches, the *on-hash* datasets [10]. This research seeks to further identify evidence in targeted micro-bloggers’ *off-hash* micro-blogs, and establish whether this evidence improves the certainty of their witnessing status. Researchers have acknowledged that event relevant micro-blogs may appear off-hash for numerous reasons, including typographic errors, ignorance of the hashtag, or omission of hashtags when engaging in direct conversations [11]. But generally, the challenges of harvesting these potentially relevant micro-blogs outweigh the benefits, particularly when there is a surplus of on-topic posts [11]. This assumption is tested by this research because posts from micro-bloggers OTG are typically a small fraction of all event relevant posts [12], and concerns of misleading and ambiguous information on social media.

Twitter case studies, Australia Football League (AFL) matches held at the Melbourne Cricket Ground (MCG) are utilized to support this research [6, 10]. Sporting events are a popular *social search* topic [13] and of interest to journalism, emergency management related to mass gatherings, and event detection and summarization research [14–16]. More particular to the evidence test processes in this study, these case studies are beneficial due to the complexity introduced by micro-bloggers who are witnessing the event live via a broadcast, described as a space-adjusting technology [17]. The research in this study presents new supervised classification experiments to demonstrate the *filter* and *extract* processes of the framework, seeking improved results from previous work [6, 10]. In particular, improvements to the precision of evidence classified OTG are required. The experiments seek to achieve these requirements by a number of enhancements to improve the training models, that include the addition of further training samples from a similar event instance and pruning atypical samples over-represented in miss-classification analysis.

This paper is structured as follows. The Background describes previous work towards identifying evidence of witnessing in micro-blogs and their characteristics, and the DST for evidence combination. The Theory Section presents original models for evidence and evidence combination. The Methodology and Results describe the methodology for implementing the evidence model to a case study event and the results. A discussion of the experimental results and conclusions complete the paper.

## Background

A review of research related to the concept of event witnessing in social media e.g. [1, 7, 9, 12, 14, 18, 19], reveals significant interest although the definition of witnessing varies in part due to differing requirements of the motivating application domains. For example, contributions by [9] and [18] are towards distinguishing micro-blogs from the wider geographic area in which the event occurs rather than direct observations of the event, a distinction essential for journalistic applications [7]. And the interest of [14] and [16] is event detection, that does not seek to distinguish spectators who are OTG from those watching on television. The previous body of work by the authors [3, 5, 6, 10] is more aligned with the recent work by [7] and [19], where the interest is distinguishing direct observations or experiences of the event. But in comparison, the research presented in this study makes unique contributions by seeking evidence and counter-evidence from the image content of micro-blogs in addition to text or geotags. And significantly, the evidence is combined to test a micro-blogger’s witnessing status in addition to individual micro-blogs. Further the case study selected can be differentiated from the typical crisis events selected e.g. [1, 7, 9, 12, 18, 19].

### Previous content descriptions towards evidence

The majority of content to be adopted as evidence in this research, has been introduced incrementally in previous work but not formally modeled [3, 6, 10]. WA and Impact Accounts (IA) were defined from the study of text and image content of micro-blogs (see Table 1), and numerous case studies were undertaken to test these definitions [5]. Both WA and IA support inferences the micro-blogger who posted them is a witness to the event, however, an important distinction is a micro-blog can only be categorized as a WA if it contains a direct observation of the event.

**Table 1:** Summary definitions of WA and IA [3, p.6].

Category	Definition	Example
**Witness Account (WA)**	A report in which a witness provides a direct observation of the event or its effects.	*…Bushfire? I can smell smoke and hear the whirlybirds right now* [20]
**Impact Account (IA)**	A report in which a potential witness describes being directly impacted or taking direct action because of the event and/or its effects.	*Had to cancel my last home visit of the day due to a bushfire* [21]
	Includes explicit declarations by a potential witness of their location in proximity to the event or its effects.	*#Bushfire that is only a 15 min drive away from my house is scary. Lucky we are East of it* [22]

**Fig 2:**
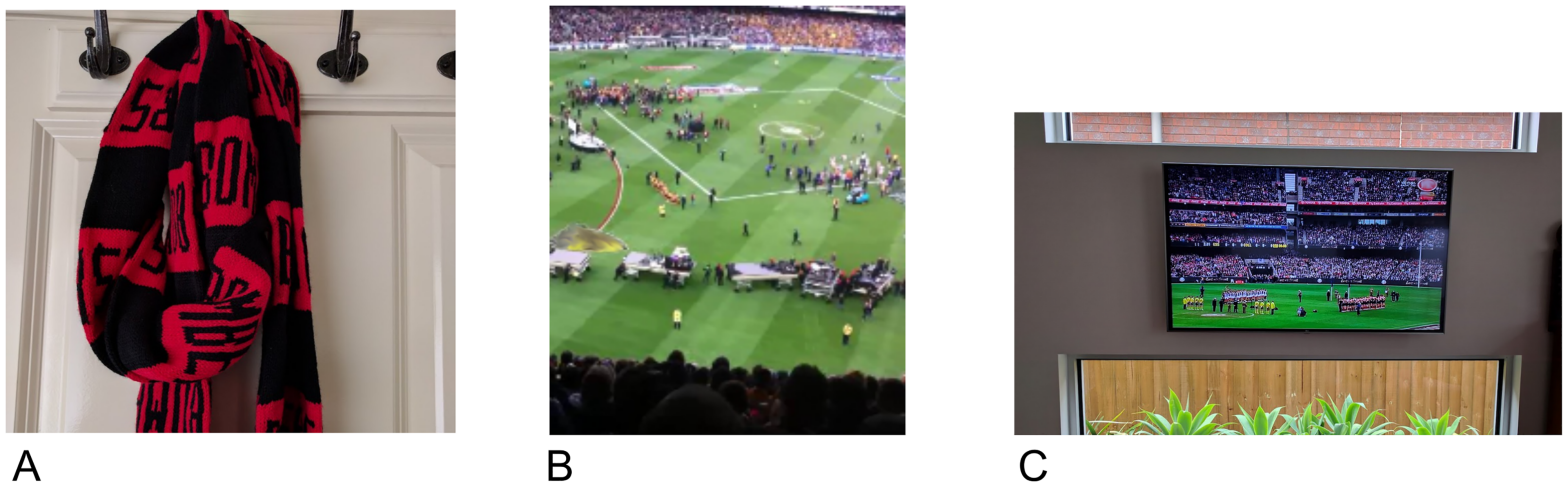
Illustrative image content to describe the evidence inference categories A) NE, B) OTG, and C) NOTG. A) and C) are printed under a CC BY license, with permission from Marie Truelove, original copyright 2017. B) printed under a CC BY license, with permission from Rachael Hopkins, original copyright 2017.

In [6], counter evidence to the witnessing status of a micro-blogger from OTG were introduced, with a description of the NOTG category. Examples from a case study event text and image content were used to describe categories of OTG, NOTG, or no evidence (NE), that are presented in Table 2 and Fig 2. As the case study event was broadcast live to a much greater audience, the inferences as to the micro-blogger’s posting location are limited unless the medium of observation or location context were explicitly stated [6, 10]. For example in Table 2 and Fig 2, presence at the event venue is clearly stated and visible in the OTG examples, and the televised broadcast in the NOTG examples.

**Table 2:** Example text content to describe the evidence inference categories NE, OTG and NOTG [6, p.3].

No Evidence (NE)	Evidence OTG (OTG)	Evidence not OTG (NOTG)
*Fletcher goes bang with a 60 metre monster! #AFLDonsPies* [23]	*Not the best seats in the house but just glad to be here at @MCG #AFLDonsPies…* [24]	*In front of TV with chips for next 3 hours! #AFLDonsPies* [25]

### Methods for fusion or combination of evidence

Previous research [6] confirmed that multiple evidence can exist within a individual micro-blog, which can either corroborate or strengthen the categorization of that micro-blog, or conflict. One conflict scenario identified was the delayed posting of text and image evidence OTG, being combined with geotags categorized NOTG because they can only reflect the location of the micro-blogger at the time of posting. The root cause of this conflict was inadequate consideration of the temporal filtering of the content sources before combination [6]. Evidence from different content sources can also have varying inferential weight [26]. This study is motivated to implement a method to combine or fuse these different content types per micro-blogger, that can both represent the varying uncertainty of the individual evidence and enable inferences as to the witnessing status of the micro-blogger.

A survey to provide an overview of state-of-the-art fusion strategies for multimedia researchers identifies that fusion of data from different modalities is generally performed at the feature level or the decision level [27]. The primary advantages of feature level fusion are that any correlation between features from the different modalities can be leveraged and there is only one learning phase. However, synchronizing time and representing features in the same format for every modality can be challenging. Advantages of decision level fusion include decisions are typically represented in the same format in comparison to feature representations of different modalities. Additionally, it allows the most suitable method of feature extraction for each modality to be deployed [27].

Methods for fusion can be categorized as rule, classification or estimation based [27]. Widely adopted rule-based methods include linear weighted and majority voting methods, and estimation based methods include Kalman filter and particle fusion that are typically used for estimates of low level data. Classification based methods include Bayesian inference and Dempster Shafer Theory of Evidence (DST). Recent work related to user generated content and GIScience that incorporate DST motivate further exploration of this method [28, 29]. Research presented in [29] experimentally compare four evidence combination methods: Bayes; DST; Fuzzy Sets; and Possibility theory, to test crowdsourced land cover data sourced from the Geo-Wiki project by a geographic weighted approach. [28] implement DST to combine Twitter location data including micro-blog text and geotags to infer the geographic location of events. The precedence by [28] of combining social media content with different modalities motivate the exploration of this methodology for the implementation of the test processes in this study.

### Dempster Shafer Theory of Evidence

DST has been frequently applied to manage uncertainty and incomplete reasoning [30]. The representation of uncertainty as an interval in DST is shared by two other major frameworks: imprecise probabilities and possibility theory [31]. Interval-based approaches have been developed for situations where it is difficult to represent uncertainty as a precise probability measure, for example where information is non-specific, ambiguous or conflicting [31]. Influences that can motivate the selection of DST include a versatility to represent and combine varying evidence types from multiple sources, the advanced theoretical development, and significant number of applications adopting DST [31]. These applications include the fusion of multiple classifier results (ensemble learning) e.g. [32–34], and a diverse range of applications in GIScience e.g. [28, 29, 35, 36]. Examples of further theoretical developments or elaborations of DST include the Transferable Belief Model (TBM) [37] and more recently Dezert-Smarandache Theory (DSmT) [38].

To implement DST the set of possible solutions are modeled as the Frame of Discernment Θ, and basic probability assignments are made for subsets of Θ represented by mass functions *mf* Eq (1).

$$\begin{array}{c} mf:2^{\Theta }\to \left[0,1\right],mf\left(\theta \right)=0\;\text{and}\;\sum_{A\subseteq \Theta }mf\left(A\right)=1 \end{array}$$ (1)

The belief *Bel* and plausibility *Pl* for any subset of Θ are computed from *mf* Eq (2). The belief interval for a set *A* is *[Bel(A),(Pl(A)]*, which is interpreted as the lower and upper probability bounds.

$$Bel\left(A\right)=\sum_{\begin{array}{l} B\subseteq A\\ B\neq \emptyset \end{array}}mf\left(B\right),\;Pl\left(A\right)=\sum_{\begin{array}{l} B\subseteq \Theta \\ A\cap B\neq \emptyset \end{array}}mf\left(B\right)$$ (2)

If evidences are derived from different sources with different reliabilities, it is possible to account for these differences by applying a discount factor *df* to *mf* Eq (3).

$$\begin{array}{c} \begin{array}{c} mf_{i}^{d}f\left(A\right)=dfmf_{i}\left(A\right)\text{,}\;\forall A\neq \Theta \\ mf_{i}^{d}f\left(\Theta \right)=1-df+dfmf_{i}\left(\Theta \right) \end{array} \end{array}$$ (3)

where 0 ≤ *df* ≤ 1 is the reliability weight of source *i*.

When evidence are derived from multiple sources from the same frame of discernment, these are aggregated by the use of a combination rule. Combination rules state how two *mf* are aggregated into one *mf*. Dempsters Rule of Combination was that originally proposed Eq (4).

$$mf_{12}\left(X\right)=\frac{1}{1-K}\sum_{\begin{array}{l} A,B\subseteq \Theta \\ A\cap B=X \end{array}}mf_{1}\left(A\right)mf_{2}\left(B\right),$$ (4)

∀*X* ⊆ Θ, *X* ≠ ∅ where *K* is the *degree of conflict* between the two *mf* Eq (5).

$$K=\underset{\begin{array}{l} A,B\subseteq \Theta \\ A\cap B=\emptyset \end{array}}{\sum }mf_{1}\left(A\right)mf_{2}\left(B\right)$$ (5)

This is a conjunction rule (*and* operation) and ignores all conflict through the normalization factor *K*, which can produce counter-intuitive results notably described by [39]. Numerous combination rules have been proposed that are disjunctive (*or* operation) or trade-off (variations of both *and* *or* operations) which do not normalize conflict. These include Yager’s rule where conflict is assigned to the universal set rather than the null set [31], and more recently PCR5 and PCR6 are proposed based on the Proportional Conflict Redistribution principle (PCR) [40].

Combination rules can also be described according to algebraic properties including associativity [31]. A combination rule is associative if (*mf*_1_ ⊗ *mf*_2_) ⊗ *mf*_3_ = *mf*_1_ ⊗ (*mf*_2_ ⊗ *mf*_3_), that is the order of combination does not change the resulting *mf* [34]. (In this paper combination is represented by the ⊗ symbol, and does not represent a specific combination rule). However, with non-associative rules the order of combination does impact the resulting *mf*. The combination rules of Yager and PCR6 are non-associative, whereas Dempster rule are associative.

A decision of the most likely state is supported by the *mf*, however, this requires interpreting the interval *[Bel(A),(Pl(A)]*, which may overlap the interval of another subset [34]. A sophisticated approach for supporting decisions is a *pignistic transform* [41] to construct a probability measure from *mf*. A more direct approach is to make a decision by adopting the state with the maximum belief or plausibility. Ranking by *Bel* is an alternative approach that has been adopted where ranking of the results rather than a decision is required [28].

Modeling an application using DST can be complex [31]. Once Θ is modeled, the method of deriving the *mf* and which combination rule to adopt are influential implementation decisions. The *mf* and *df* can be derived by experts in the application domain, an approach adopted in previous research [6]. However, many implementations seek to derive a representative *mf* of the information source and process through automatic means.

Previous research [29] describe using a tri-cube kernel to compute a *mf* for each crowdsourced data point in their application. A weight *w* is calculated for each data point *P* based on its distance to the centre of the kernel as follows:

$$\begin{array}{c} w_{ij}=1-({\left(d_{ij}\right)}^{3}/b^{3}) \end{array}$$ (6)

where *d_ij_* is the distance in meters from the center of the kernel *K_i_* to the crowdsourced data point *P_j_*, and *b* is the bandwidth at that location. The resulting weight *w* is adopted as the *mf* for the class declared by the contributor at this location, and 1 − *w* is attributed to the remainder hypothesis declared by Θ. This has the desired effect of producing a *mf* with greater belief close to the center of the kernel. An adapted version of this approach may be more appropriate than the decision boundaries implemented in preliminary experimentation [6].

Research has employed many methods for the combination or fusion of classifier results including DST, and there are a number of approaches to derive *mf* when employing DST [42], including using the confusion matrix result [32]. The frame of discernment for a classifier can be defined as Θ_*C*_ = {*θ*_*c*_|*c* ∈ *C*} where *C* is a set of classes and *θ*_*c*_ represents the hypothesis that a new sample is of class *c*. The recognition rate *ε*_*r*_ of the proposition class *c* ∈ *C*, is assigned as the *mf* for samples of that class *mf*(*θ*_*c*_). And the substitution rate *ε*_*s*_ to the complement of *θ*_*c*_, that is *mf*(¬*θ*_*c*_). The recognition rate for a class *c* is the ratio of the number of samples classified as the class *c* to the total number of samples presented to the classifier of belonging to class *c*. Research in [42] alternatively propose the predictive rate *ε*_*p*_, which is defined as the ratio of the number of input samples classified correctly to class *c* to the total number of samples classified as class *c*.

## Theory

This section defines models to represent and test evidence.

### Evidence

Content found in micro-blogs can only be defined as evidence if it supports an inference the micro-blogger who posted it is OTG or NOTG. This inference is either direct, that is a declaration of the location of the micro-blogger, or because the micro-blogger is a potential witness by some other evidence. To be a witness of an event or its effects the micro-blogger has to be OTG or in close proximity [3]. Table 3 lists on-topic content that qualifies as evidence [3, 6], the inferential assumptions this evidence supports, and qualifies if this evidence type can be categorized as a WA or IA.

**Table 3:** A summary of evidence types for on-topic micro-blogs.

Content	Type of evidence *Example*	Micro-blogger Inferences	WA/ IA
Micro-blog text	Direct effect observation topic *I see smoke*	Witness ∴ OTG	WA
	Direct impact or action topic *We are evacuating*	OTG ∴ Witness	IA
	Declaration OTG *I’m at the MCG*	OTG ∴ Witness	IA
	Anticipated attendance OTG *I’m on my way to the MCG*	OTG ∴ Witness	-
	Declaration NOTG *I wish I was at the game*	NOTG ∴ not Witness	-
	Declaration via broadcast *Watching the game on TV*	Witness via broadcast ∴ NOTG	-
Linked image	Observation of event	Witness ∴ OTG	WA
	Observation of event broadcast	Witness via broadcast ∴ NOTG	-
Linked geotag	Posted at event	OTG ∴ Witness	-
	Not posted at event	NOTG ∴ not Witness	-

The evidence summary includes the micro-blog content source, the inferences the evidence supports, and whether this evidence qualifies as a WA or IA.

#### Evidence in off-hash datasets

All the evidence types listed in Table 3 have resulted from the study of on-topic micro-blogs, meaning they are related to the target event. A sample of on-topic micro-blogs are typically distinguished using event specific hashtags or keywords resulting in the terminology *on-hash* datasets [10]. The search micro-blogger processes of the framework introduced in Fig 1, are an exploration of the off-hash dataset for micro-bloggers with evidence identified on-hash. The evidence types presented in Table 3 may be present in the off-hash dataset of a micro-blogger, as previously described, not all on-topic micro-blogs will be present in the on-hash datasets [11]. The pragmatic place to begin the search of further evidence in the off-hash datasets is the on-topic content because their characteristics have already been described.

**Fig 3:**
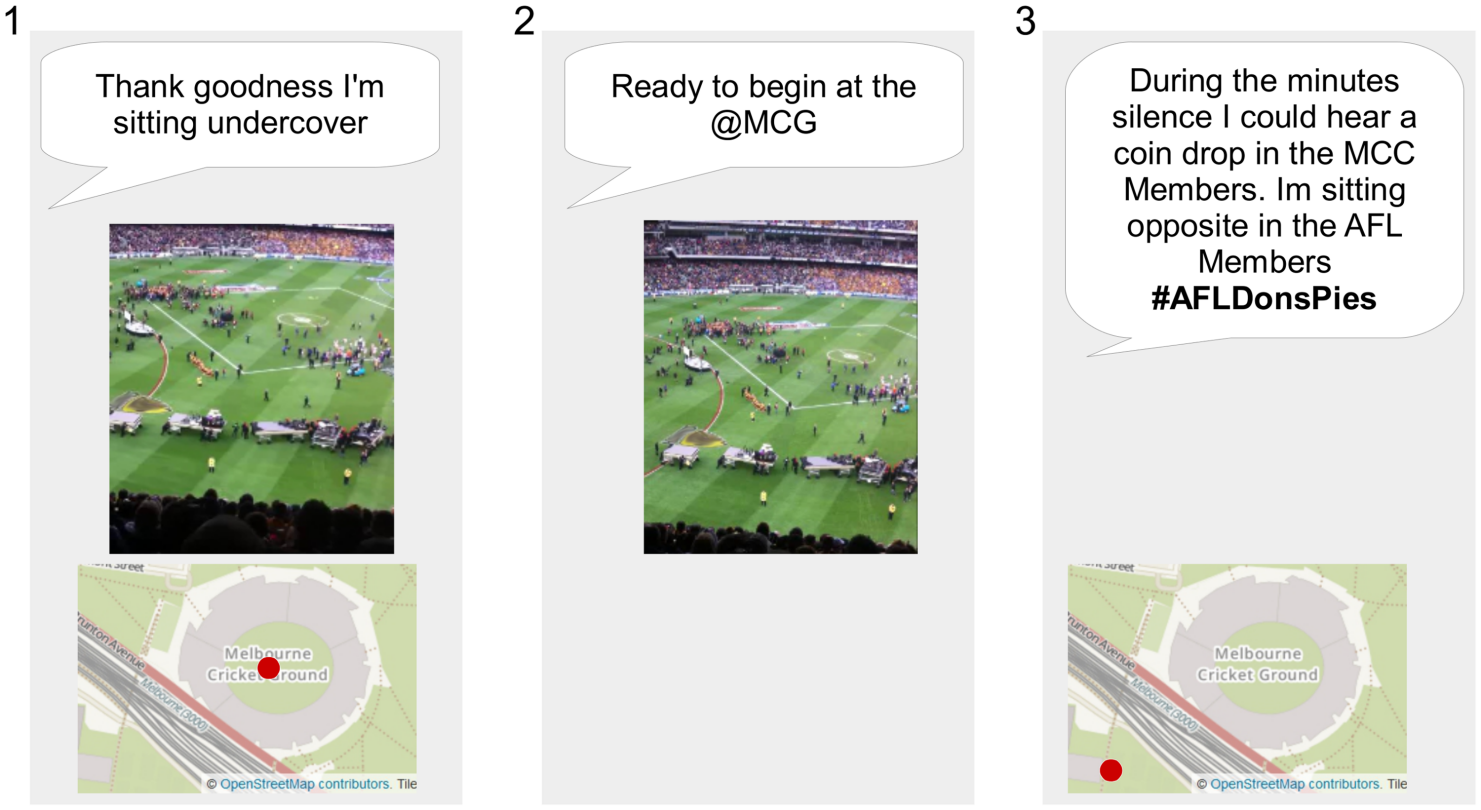
Illustrative example of on-topic micro-blogs posted by a single micro-blogger that distinguish between on-hash and off-hash categories for a target event *#AFLDonsPies*. The image and text content in this figure are similar to content posted by an example micro-blogger, and are for illustrative purposes. The images in the figure are printed under a CC BY license, with permission from Rachael Hopkins, original copyright 2017.

Fig 3 presents example micro-blogs to highlight this concept. Micro-blog number three contains the hashtag *#AFLDonsPies* related to the target event, and therefore, can be described as both on-topic and on-hash. Inspection of the content of micro-blog number 3 also reveals conflicting evidence, text content which supports the inference the micro-blogger is at the event, however, a geotag which is located outside the event venue. A search for further on-topic micro-blogs in this micro-blogger’s time-line reveals two earlier posts, micro-blog one and two. They were not detected in the initial search as they do not contain the hashtag, they are off-hash, however, do contain further evidence the supports the hypothesis that the micro-blogger is OTG at the event.

Other evidence types in the off-hash datasets can be predicted, for example text or image content that indicates a micro-blogger is posting from a different event to the target event. Exploration of these off-topic evidence are considered outside scope of this research currently, with exception of text where the micro-blogger explicitly states their presence at some other event, such as the examples in Table 4. These micro-blogs were posted by micro-bloggers who were posting content on-hash about the target event *#AFLDonsPies* similar to the micro-blogger in Fig 3. However, the search in their off-hash content reveals content related to multiple events and explicitly locates them at one of these events.

**Table 4:** Examples of potential NOTG evidence from the off-topic text content.

Example text
a) *Here @ManukaOval & ready to cheer for the mighty @GWSGIANTS. #AFLGiantsSuns #HereComeTheGiants #IBelieveThatWeWillWin* http://t.co/HtRgRM84Nu [43]
b) *Poling the banner in damp Canberra. #AFLGiantsSuns #HereComeTheGiants* http://t.co/y73QtC8OKN [44]
c) *@CarltonFC fantastic win. Well worth the trip from Sydney. Even got pat on the back from the Board #AFLSaintsBlues* http://t.co/yhEXI15QNt [45]

Example micro-blogs that explicitly locate the micro-blogger at an event that is not the target event *#AFLDonsPies*.

#### Uncertainty of evidence

A characteristic of the evidence types presented in Table 3 is the inferences they support are of varying uncertainty. This research does not claim to document all aspects of uncertainty, but aims to identify the influences on variation to enable informed consideration with new event types and instances. The influences identified are the content source, the processes undertaken to filter and extract the evidence, and the inferential weight of the evidence towards the hypothesis of OTG or NOTG. The influences are now discussed for each currently identified content source.

Geotags represent point locations linked to micro-blogs, often sourced from technologies such as GPS. The limitations of GPS are common knowledge and include restricted indoor application. It is necessary to assume a micro-blogger is mobile and therefore, a geotag only represents a micro-blogger’s location for a moment corresponding to the timestamp. Unlike images and text, geotags do not require complex filtering and extraction processes to identify content which support inferences of OTG or NOTG. To determine which, the spatio-temporal characteristics of the event and the scale of expected geotag error need modeling. These can vary significantly, for example an event confined to a stadium compared to the scale of a cyclone. The inferential weight of the geotag as to the location of the micro-blogger at posting is strong, however, not absent from limitations. For example location spoofing [46] raises the possibility of intentionally misleading geotags.

Micro-blog text content is available with every post and limited by character length, for example tweets of 140 characters [47]. The text content can represent human thought displaced from the micro-blog’s timestamp. Observations and places experienced in the past, present, and anticipated future can be included. Descriptions can range from spatially and temporally precise, to vague, to intentionally misleading. Extensive filtering and extraction processes are necessary to identify the small fraction of available text that can be considered evidence. Each filtering and extraction process can introduce error that can be described, for example the confusion matrix resulting from supervised classification. As a consequence of these characteristics, a single piece of text evidence may not be considered decisive in most circumstances. The inferential weight is weak relative to geotags and variable, due to the variability of individual micro-blogger’s reports for example.

The micro-blogger has freedom to link images from any source, from photographs they have just taken of the event, to an archived animation. All image meta-data are removed when posted to many social networks [48], meaning it is not available to verify the source, time, or location of capture. However, generally images of real-life scenes can be considered more informative and less subjective relative to text [49], and therefore, considered to have greater inferential weight. Additionally, previous case studies indicate the proportion of linked images that are distinguished as evidence are relatively high compared with text [5]. The inferential weight may vary, as the target of the images may vary. For example, images depicting queues outside a venue are less compelling than images of the event underway inside. Images of an event cannot be captured until the event is in progress, but posting of these images can be delayed. Similar to text, extensive filtering and extraction processes are necessary to identify image evidence.

#### The variability of uncertainty due to event characteristics

The uncertainty associated with each evidence type can vary with the characteristics of different event types and instances. These event characteristics may impact a whole content source, for example geotags, or just a particular evidence type. The variability may not be significant, or can completely negate the inferential weight of a type of evidence. Each source of uncertainty described in the previous Uncertainty of evidence Section, for each evidence type described in Table 3 must be considered with respect to each new event scenario. There are many influences on event scenarios, however, the most consequential identified are the spatial and temporal characteristics of the event [5].

#### Combining evidence

The evidence is combined to test whether they corroborate the hypothesis the micro-blogger is OTG or NOTG. A corroboration result represents a reduction in categorization uncertainty, a conflict result calls the micro-blogger’s status into question. Previous research indicates that conflict is more likely due to categorization errors introduced during the filtering and extraction processes rather than fake or malicious content [6]. For example the varying spatial and temporal characteristics of geotags compared to the flexibility of images and text can cause conflict [6]. One approach to reduce this conflict is filtering geotags to the time interval of the event compared with images and text which may include before and after the event.

This research also proposes that conflict and corroboration within a micro-blog may be interesting to distinguish between conflict and corroboration between micro-blogs sourced from a single micro-blogger. Micro-blog number three in Fig 3 is an example of conflict within a single micro-blog, whereas micro-blog one and two in Fig 3 are multiple micro-blogs corroborating each other. In addition to the same categorization errors that result in conflict within micro-blogs, conflict between micro-blogs may represent a legitimately different categorization of the micro-blogger. For an event of scale beyond the vista [50], for example a cyclone, the micro-blogger may legitimately post evidence OTG and NOTG. This highlights the importance of temporal and spatial filters or windows for evidence combination, defined with consideration of the spatial and temporal characteristics of the event.

### A set representation of evidence

Evidence is formally modeled to abstract concepts from implementation methodologies. A formal model can therefore support the development of multiple test implementations, accommodate the introduction of new evidence types, and be adaptable for different event types.

A stream of micro-blogs can be searched to discover those relevant to an event. Each micro-blog contains meta-data including a timestamp *ts* of posting, a unique identifier *mid*, and a unique identifier of the micro-blogger *uid*. Ordered micro-blogs can be assigned to each micro-blogger based on the *uid*.

A set of multiple content sources *S* can be defined for micro-blogs including the micro-blog text content *T*, optional geotags *G*, and the optionally linked images *I*. Other content sources may be defined, including sub-categories of each content source, for example, the text content source *T* can be split into {*T*_1_, *T*_2_,…,*T*_*q*_} to model different uncertainties for different evidence types (see Table 3).

As multiple content sources can be associated with each micro-blog, a set of evidence is defined *M* = {*e*_1_, *e*_2_,…,*e*_*n*_}. Evidence can additionally can be assigned to a micro-blogger by the inherited *uid* directly *B* = {*e*_1_, *e*_2_,…,*e*_*m*_} or maintaining micro-blog boundaries *MB* = {*M*_1_, *M*_2_,…,*M*_*p*_}.

Each piece of evidence is assigned a single inference category of the status of the micro-blogger *C* = {*OTG*, *NOTG*, *NE*}. If the evidence does not support the inference categories *OTG* or *NOTG*, it is said to provide no evidence (*NE*). *NE* is necessary because the filter process is imperfect: it is not possible to eliminate all content that does not support inferences of witnessing by the filtering process.

For inference testing purposes a set of evidence *MT* is defined as the subset of *M* containing evidence that intersects *OTG* or *NOTG*. Similarly, the set *MBT* is defined as the subset of *MB*. Evidence of inference category *NE* is not included because it does not conflict or corroborate the status of the micro-blogger.

**Fig 4:**
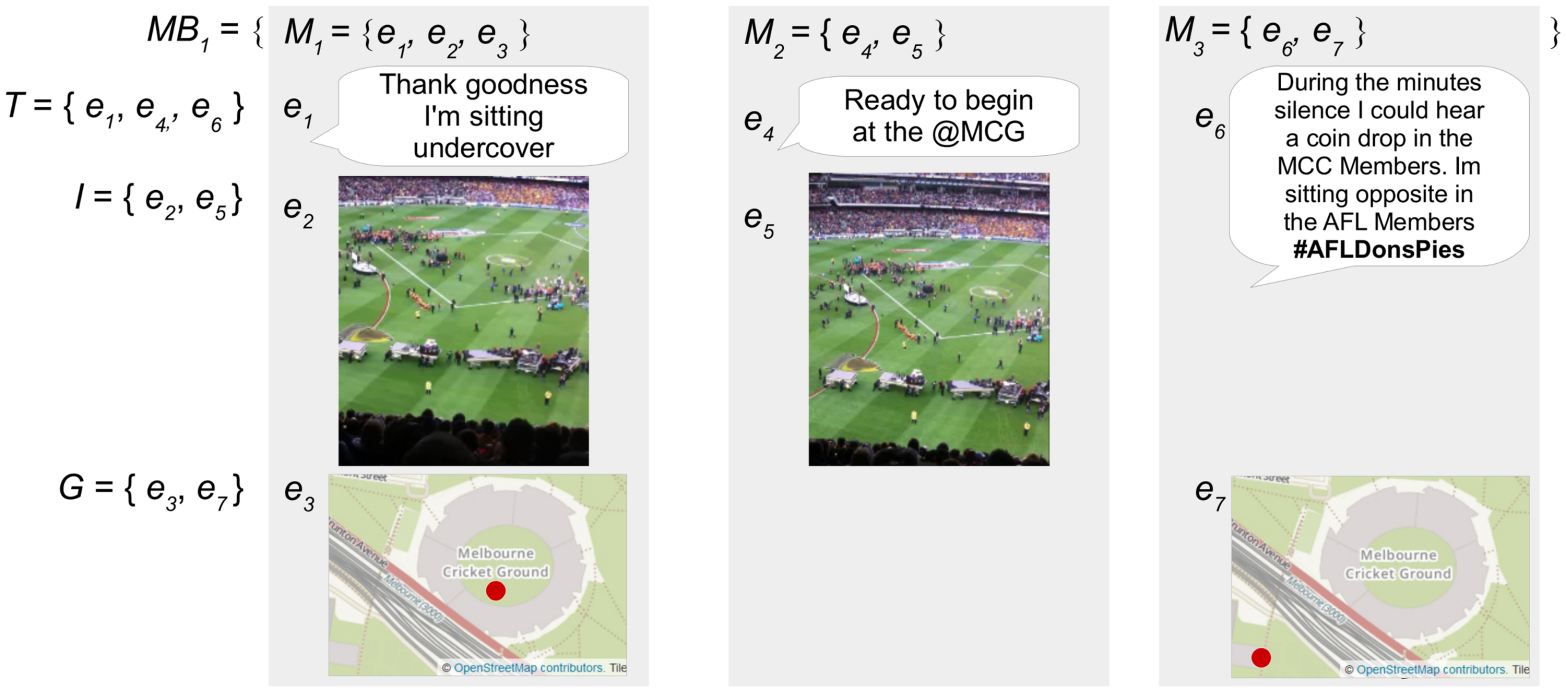
The sets representing evidence and micro-blogs posted by an example micro-blogger. The micro-blogger *MB*_1_ has posted three micro-blogs *M*_1–3_ that include three text evidence *T* = {*e*_1_, *e*_4_, *e*_6_}, two images *I* = {*e*_2_, *e*_5_}, and two geotags *G* = {*e*_3_, *e*_7_}. The image and text content in this figure are similar to content posted by an example micro-blogger, and are for illustrative purposes. The images in the figure are printed under a CC BY license, with permission from Rachael Hopkins, original copyright 2017.

**Fig 5:**
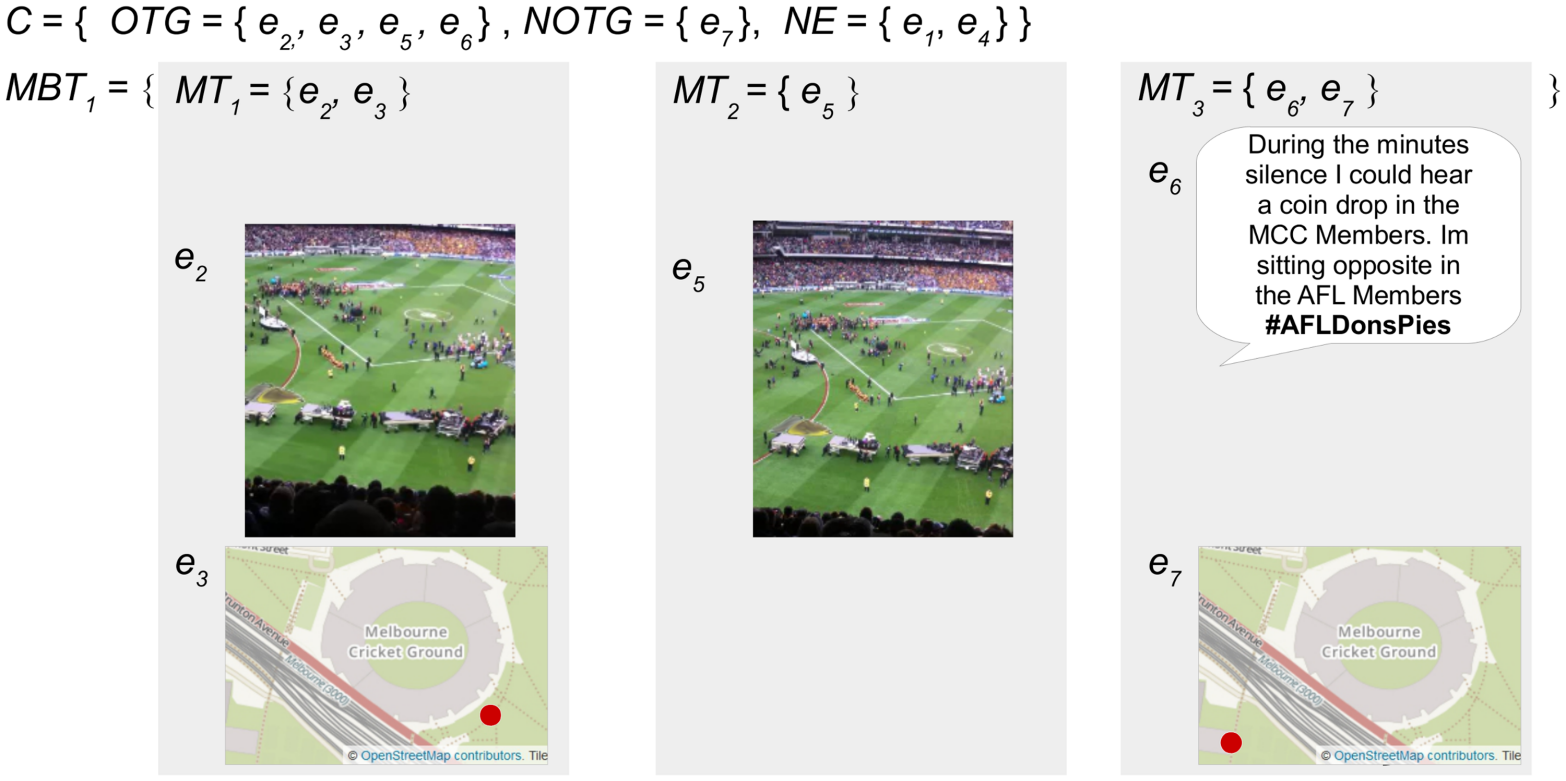
The subsets of evidence defined for testing the example micro-blogger. Five pieces of evidence *e*_2, 3, 5–7_ categorized as *OTG* or *NOTG* remain for inference testing within three micro-blogs *MT*_1–3_ after content categorized as *NE* is removed. The image and text content in this figure are similar to content posted by an example micro-blogger, and are for illustrative purposes. The images in the figure are printed under a CC BY license, with permission from Rachael Hopkins, original copyright 2017.

Fig 4 presents the defined sets for an example micro-blogger *MB*_1_ who has posted three micro-blogs related to an event. The micro-blogger *MB*_1_ has posted three micro-blogs *M*_1–3_ that include seven pieces of evidence *e*_1–7_ from text, image and geotag content sources. Fig 5 demonstrates the subsets of evidence that are categorized as *OTG* or *NOTG* for inference testing.

#### Derivation of micro-blog combined inference category

**Fig 6:**
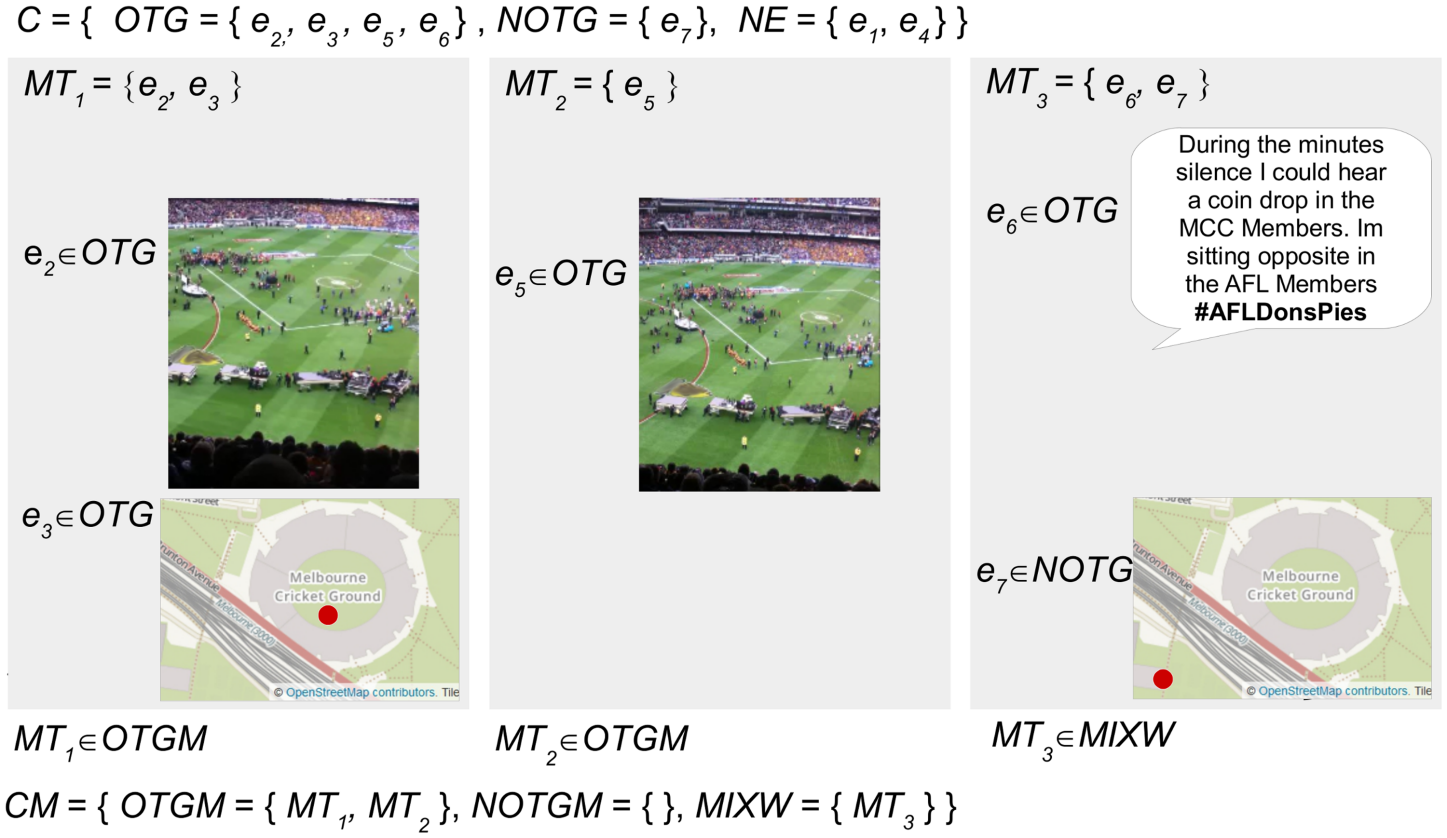
The derivation of the combined inference category for each micro-blog posted by the example micro-blogger. The micro-blogs *MT*_1_ and *MT*_2_ are categorized *OTGM* because all evidence contained are categorized *OTG*. *MT*_3_ is categorized *MIXW* because it contains evidence that are both *OTG* and *NOTG*. The image and text content in this figure are similar to content posted by an example micro-blogger, created for illustrative purposes. The images in the figure are printed under a CC BY license, with permission from Rachael Hopkins, original copyright 2017.

As demonstrated in Fig 5 a micro-blog can contain multiple evidence. Each micro-blog is assigned a combined inference category based on the inference categories of these evidence. This derivation is demonstrated in Fig 6 for the example micro-blogger *MB*_1_. *MT*_3_ is assigned the combined inference category *MIXW* because it contains both *OTG* and *NOTG* evidence, whereas *MT*_1_ and *MT*_2_ are assigned *OTGM* because they contain only *OTG* evidence.

Formally, *MT* is assigned membership of one combined inference category *CM* = {*NOTGM*, *OTGM*, *MIXW*} by Algorithm 1. *MT* is a member of *NOTGM* if all contained evidence intersect *NOTG*, or *OTGM* if all contained evidence intersect *OTG*, or *MIXW* if contained evidence intersect both *OTG* and *NOTG*.

#### Derivation of micro-blog test within category

**Fig 7:**
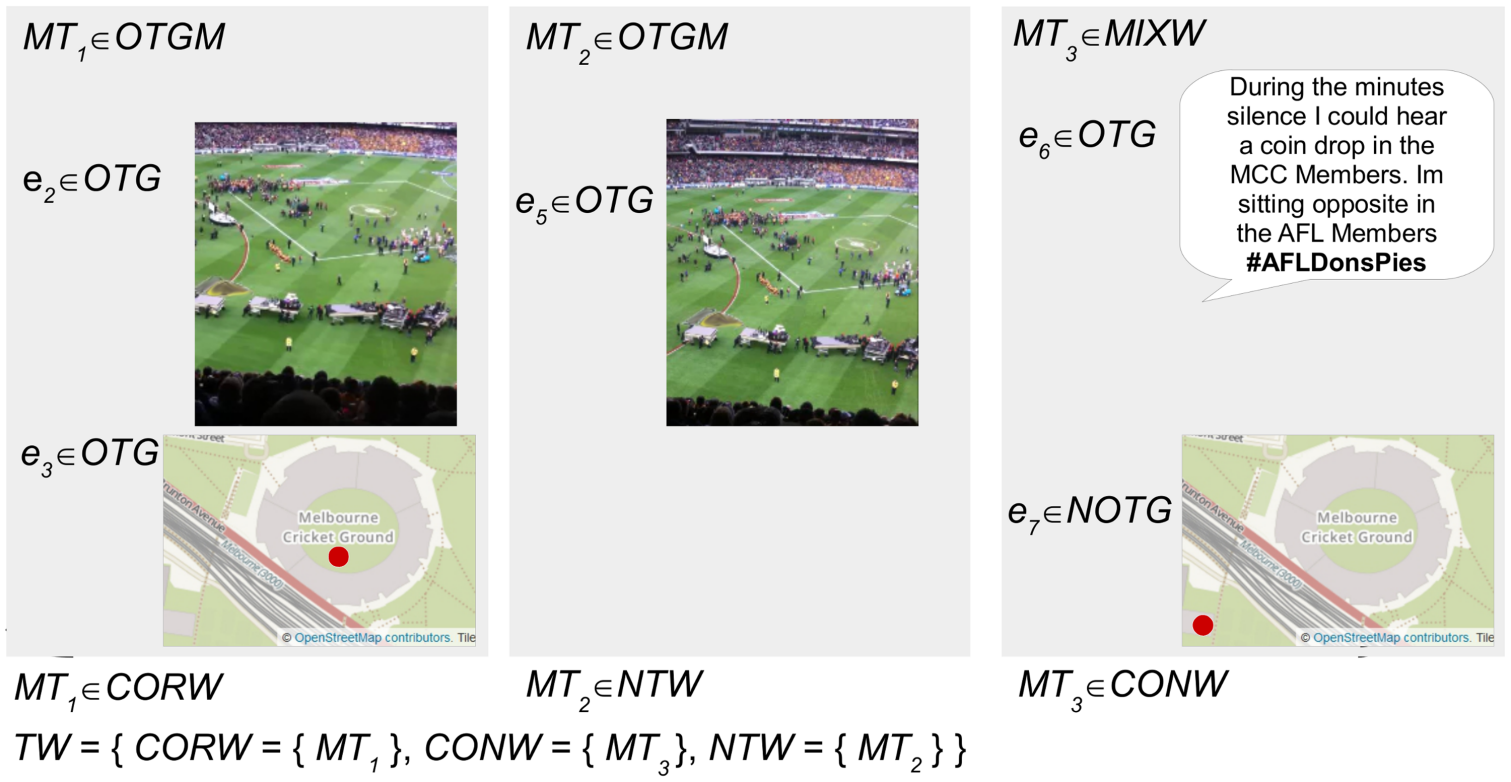
The derivation of the test within result for each micro-blog posted by the example micro-blogger. The test within result for *MT*_1_ is corroboration *CORW*, and the result for *MT*_3_ is conflict *CONW*. *MT*_2_ must be assigned no test within *NTW*. The image and text content in this figure are similar to content posted by an example micro-blogger, and are for illustrative purposes. The images in the figure are printed under a CC BY license, with permission from Rachael Hopkins, original copyright 2017.

The evidence within each micro-blog is tested for corroboration or conflict. Fig 7 demonstrates that each of the example micro-blogs has a different test result for the example micro-blogger. The test within result for *MT*_1_, with two evidence *OTG*, is corroboration *CORW*. The test within result for *MT*_3_ is conflict *CONW* because the two contained evidence are of conflicting categories. And finally, because *MT*_2_ has only one piece of evidence a test is not supported, and it is assigned the no test within category *NTW*.

Formally *MT* is assigned membership of one test within category *TW* = {*CORW*, *CONW*, *NTW*} by Algorithm 2. If *MT* contains one evidence it is a member of the no test within category *NTW*. If *MT* contains greater than one evidence of the same inference category it is a member of the corroborate within category *CORW*, otherwise it is a member of the conflict within category *CONW*.

**Algorithm 1**. **Computation of combined inference category *CM* for each micro-blog *MT***.

**if** |*MT* ∩ *OTG*| ≥ 1 ∧ |*MT* ∩ *NOTG*| = 0 **then**

 *MT* ∈ *OTGM*

**else if** |*MT* ∩ *NOTG*| ≥ 1 ∧ |*MT* ∩ *OTG*| = 0 **then**

 *MT* ∈ *NOTGM*

**else**

 *MT* ∈ *MIXW*

**end if**

#### Derivation of micro-blogger summary inference category

**Fig 8:**
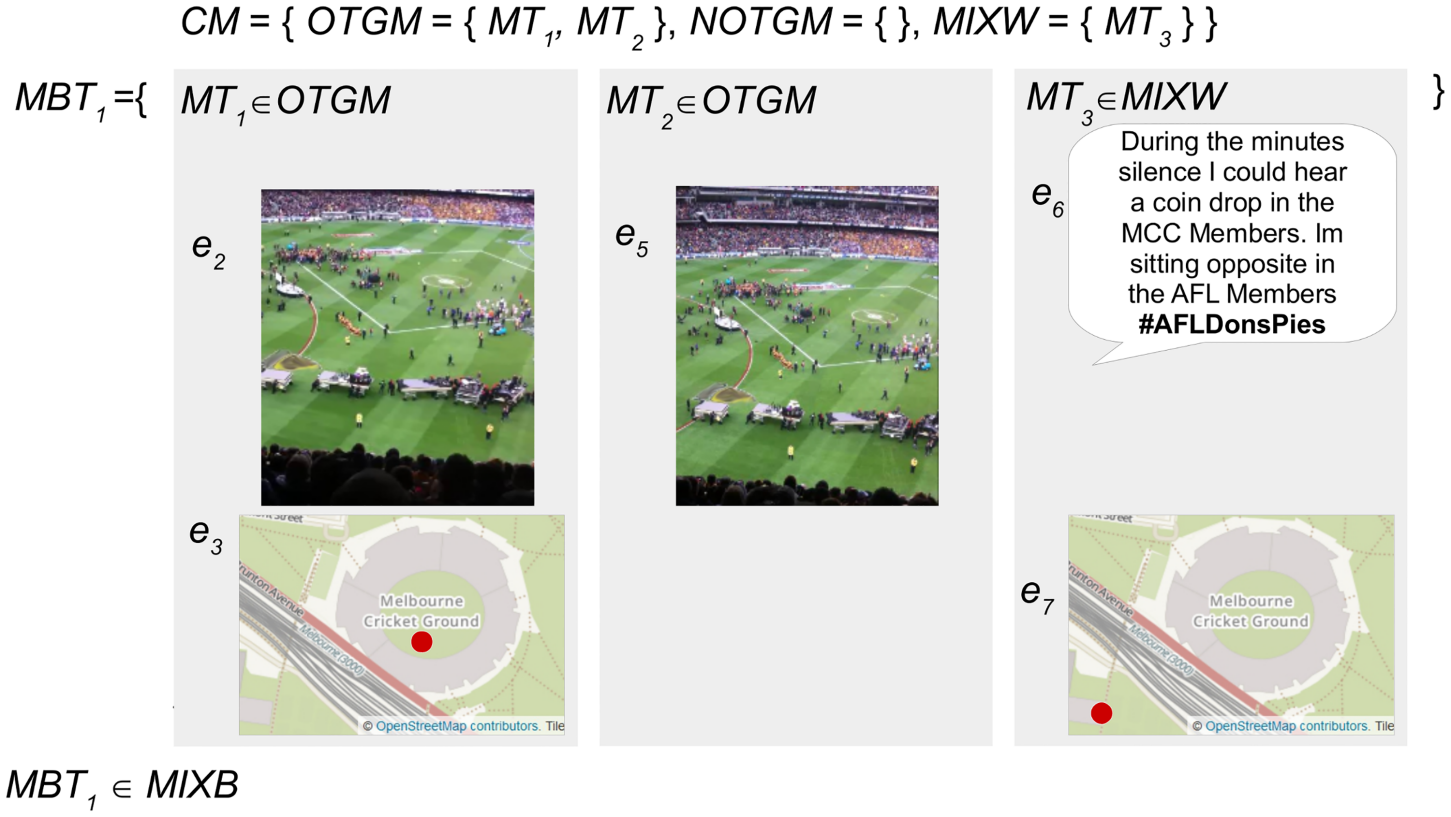
The derivation of the summary inference category for the example micro-blogger. The micro-blogger *MBT*_1_ is assigned a summary inference category of *MIXB* as they have posted micro-blogs of mixed inference categories. The image and text content in this figure are similar to content posted by an example micro-blogger, and are for illustrative purposes. The images in the figure are printed under a CC BY license, with permission from Rachael Hopkins, original copyright 2017.

Each micro-blogger is assigned a summary inference category derived from the combined inference categories of the micro-blogs they have posted. Fig 8 demonstrates that the summary inference category for the example micro-blogger is *MIXB*, because the micro-blogs they have posted are of varying inference categories, specifically *MT*_3_ is *MIXW*.

Formally, *MBT* is assigned membership of one summary inference category *CB* = {*OTGB*, *NOTGB*, *MIXB*} by Algorithm 3. *MBT* is a member of *OTGB* if all member *MT* intersect *OTGM*, or *NOTGB* if all member *MT* intersect *NOTGM*, otherwise *MBT* is a member of *MIXB*.

**Algorithm 2**. **Computation of test within category *TW* for each micro-blog *MT***.

**if** *MT* ∈ *MIXW* **then**

 *MT* ∈ *CONW*

**else if** (*MT* ∈ *OTGM* ∨ *MT* ∈ *NOTGM*) ∧ |*MT*| > 1 **then**

 *MT* ∈ *CORW*

**else**

 *MT* ∈ *NTW*

**end if**

**Algorithm 3**. **Computation of summary inference category *CB* for each micro-blogger *MBT***.

**if** |*MBT* ∩ *OTGM*| ≥ 1 ∧ |*MBT* ∩ *NOTGM*| = 0 ∧ |*MBT* ∩ *MIXW*| = 0 **then**

 *MBT* ∈ *OTGB*

**else if** |*MBT* ∩ *NOTGM*| ≥ 1 ∧ |*MBT* ∩ *OTGM*| = 0 ∧ |*MBT* ∩ *MIXW*| = 0 **then**

 *MBT* ∈ *NOTGB*

**else**

 *MBT* ∈ *MIXB*

**end if**

#### Derivation of test between micro-blog category

**Fig 9:**
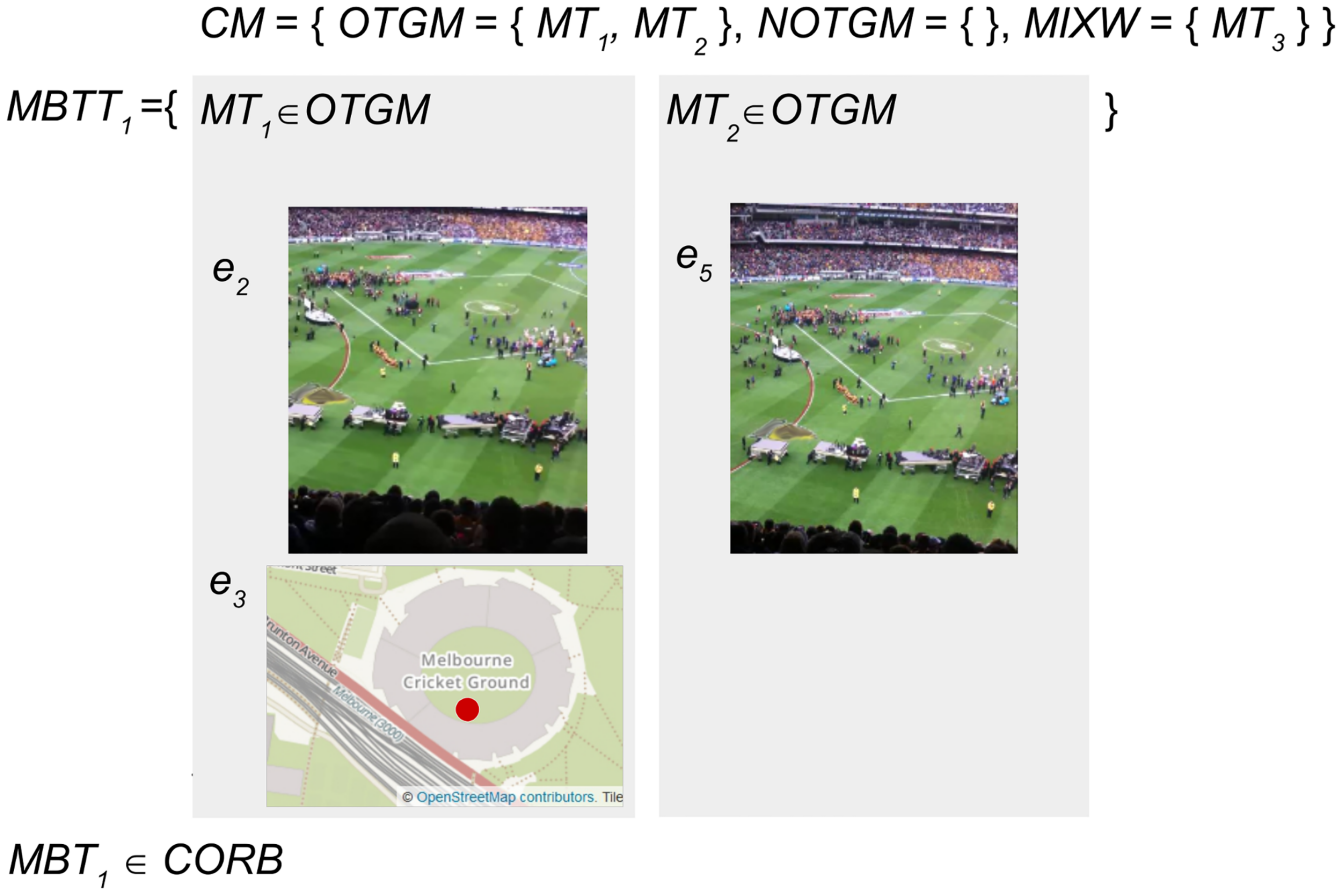
The derivation of the test between category for the example micro-blogger. Micro-blogger *MBT*_1_ is assigned the test between category corroboration *CORB* as the test set *MBTT*_1_ contains more than one micro-blog of the same combined inference category *OTGM*. The image and text content in this figure are similar to content posted by an example micro-blogger, created for illustrative purposes. The images in the figure are printed under a CC BY license, with permission from Rachael Hopkins, original copyright 2017.

The example micro-blogger *MBT*_1_ demonstrates a scenario where although conflict is detected within a single micro-blog, corroboration can exist between micro-blogs posted by a single micro-blogger. As shown in Fig 9, if *MT*_3_ with mixed inference categorization is removed, the two remaining micro-blogs *MT*_1_ and *MT*_2_ are the same inference category *OTGM*, and therefore, *MBT*_1_ is assigned the test between result of corroboration *CORB*. The primary purpose for this categorization is to identify those micro-bloggers with corroborating evidence between micro-blogs, despite conflict within a single micro-blog that may be caused by erroneous evidence categorization.

Formally, *MBT* is assigned membership of one test between category *TB* = {*CORB*, *CONB*, *NTB*} by Algorithm 4. Algorithm 4 defines a new set *MBTT* as the subset of *MBT* with members intersecting *MIXW* removed. This is necessary because members of *MIXW* cannot be said to corroborate or conflict with another micro-blog. *MBT* is a member of the corroborate between category *CORB*, if greater than one member of *MBTT* intersect *OTGM* or *NOTGM*. *MBT* is a member of the conflict between category *CONB*, if members of *MBTT* intersect both *OTGM* and *NOTGM*. *MBT* is a member of the no test between category *NTB* if *MBTT* has only one member.

**Algorithm 4**. **Computation of test between category *TB* for each micro-blogger *MBT***.

**if** 0 ≤ |*MBTT*| ≤ 1 **then**

 *MBT* ∈ *NTB*

**else if** |*MBTT* ∩ *OTGM*| > 1 ∧ |*MBTT* ∩ *NOTGM*| = 0 **then**

 *MBT* ∈ *CORB*

**else if** |*MBTT* ∩ *OTGM*| = 0 ∧ |*MBTT* ∩ *NOTGM*| > 1 **then**

 *MBT* ∈ *CORB*

**else**

 *MBT* ∈ *CONB*

**end if**

where *MBTT* = *MBT*\(*MBT* ∩ *MIXW*)

#### Derivation of micro-blogger summary corroborate status

The final test provides a summary of whether a micro-blogger has corroborating evidence without conflict, regardless of whether corroboration is from evidence within a single micro-blog (*CORW*) or from multiple micro-blogs (*CORB*). The example micro-blogger *MBT*_1_ cannot be assigned a member of *COR*, as although identified to have corroboration between micro-blogs with evidence (see Fig 9), conflict has been detected within a micro-blog (see Fig 7).

Formally, membership of *COR* indicates a micro-blogger has corroborating evidence without conflict and is computed by Algorithm 5. If *MBT* is a member of *CORB* or contains a member that intersects with *CORW*, it can be assigned a member of *COR*, unless a member additionally intersects with *MIXW*.

**Algorithm 5**. **Computation of summary corroboration category *COR* for each micro-blogger *MBT***.

**if** |*MBT* ∉ *MIXB*| ∧ (*MBT* ∈ *CORB* ∨ |*MBT* ∩ *CORW*| > 0) **then**

 *MBT* ∈ *COR*

**else**

 *MBT* ∉ *COR*

**end if**

### Evidence combination using DST

The frame of discernment Θ for evidence Eq (7) is defined from the inference categories of *C*.

$$\begin{array}{r} \Theta =\{\{\},\left\{OTG\right\},\left\{NOTG\right\},\left\{NE\right\},\left\{OTG,NOTG\right\},\left\{OTG,NE\right\},\\ \left\{NOTG,NE\right\},\left\{OTG,NOTG,NE\right\}\} \end{array}$$ (7)

Evidence is modeled by a *mf* to reflect the uncertainty of the different content sources *S* = {*T*, *G*, *I*}, and their inferential weight. The *mf* can be manually set by experts, or can be derived from the automatic process implemented to extract an evidence type and a *df* to reflect the relative inferential weight between the evidence types. Once the *mf* are derived, if *MBT* has multiple evidence, their *mf* are combined. The order of combination is informed by the set *MBT*, that is the order of posting according to the timestamps, and if there are multiple evidence for a single micro-blog this evidence is combined first. The set *MBT* is selected rather than *BT* to maintain micro-blog boundaries, as can be compared by the representations in Eqs (8) and (9) respectively. Whether combination informed by *MBT* will produce different results compared to *BT* is dependent on whether the combination algorithm used is associative or non-associative, and the structure of evidence posted with respect to micro-blog boundaries.

$$\begin{array}{c} \begin{array}{cc} & mf_{MBT}=mf_{MT_{1}}⊗mf_{MT_{2}}⊗\cdots ⊗mf_{MT_{p}}\text{,}\\ & \text{where}\;mf_{MT_{i}}=mf_{e_{1}}⊗mf_{e_{2}}⊗\cdots ⊗mf_{e_{n}} \end{array} \end{array}$$ (8)

$$\begin{array}{c} mf_{BT}=mf_{e_{1}}⊗mf_{e_{2}}⊗\cdots ⊗mf_{e_{m}} \end{array}$$ (9)

The combination of evidence for example micro-blogger *MBT*_1_ is presented in Eq (10).

$$\begin{array}{c} mf_{MBT_{1}}=mf_{MT_{1}}⊗mf_{MT_{2}}⊗mf_{MT_{3}}=\left(mf_{e_{2}}⊗mf_{e_{3}}\right)⊗\left(mf_{e_{5}}\right)⊗\left(mf_{e_{6}}⊗mf_{e_{7}}\right) \end{array}$$ (10)

## Methodology

This section first describes the search, filter, and extract processes undertaken to identify evidence, followed by the DST implementation for testing this evidence.

### Case study event

Table 5 describes two Australian Football League (AFL) events held at the Melbourne Cricket Ground (MCG), both case studies that have supported previous research [6, 10]. The Grand Final event is used solely for the supervised classification experiment.

**Table 5:** A summary of the case study events [10].

Match	ANZAC Day: Essendon vs Collingwood	Grand Final: West Coast vs Hawthorn
**Date**	Saturday 25th April 2015	Saturday 3rd October 2015
**Game Time**	14:40–17:00	14:40–17:00
**Weather**	Overcast, Rain	Sunny
**Location**	MCG	MCG
**Attendance**	88,398 [51]	98,633 [52]
**TV Ratings**	1.298M [53]	3.524M [53]

Times are in Australia Eastern Standard Time.

### Data collection

The methodology employed to collect the raw case study datasets is undertaken in stages and described previously [6, 10]. The first stage is to collect a sample of on-topic micro-blogs using the promoted hashtags for the event and the Twitter Streaming API [54]. At the events completion the second stage is commenced. The collected micro-blogs are analyzed to establish a sample of micro-bloggers who have posted at least one original micro-blog during the event. The Twitter Search API [55] are then used to collect all micro-blogs posted by these micro-bloggers, in the hours preceding the event and during the event until shortly after completion. The final stage is to analyze the linked content for each micro-blog and collect images posted from Twitter and Instagram.

To clarify, all micro-blog data was collected directly by the authors of the study in adherence to Twitters terms of use. Software tools by Twitter Data Analytics [56] were configured and deployed to access the Twitter APIs by the authors to collect the micro-blog data used by this study. The authors did not receive the Twitter datasets from another source, nor redistribute the datasets collected to support the study.

### Filter

Once collected, filtering using automatic and semi-automatic methods are employed to remove content that cannot support or test inferences of witnessing. For example all micro-blogs discovered to be retweets or contain non-original linked image content are removed [6, 10]. All micro-blogs linked to accounts promoted as companies are also removed including the AFL clubs and mainstream media [6, 10]. The micro-blog datasets are then separated into the on-hash and off-hash datasets labeled *ADon* and *ADoff* respectively.

In comparison to previous research [6, 10] additional temporal filtering was implemented. Table 6 presents a summary of temporal milestones for the case study event, and Table 7 presents the temporal filters applied. The geotags are sampled more restrictively to the time interval of the event and preceding entertainment, as are all content sources for the ADoff dataset, a conservative approach. The ADoff dataset is also filtered to those micro-bloggers with evidence discovered in the ADon dataset.

**Table 6:** A summary of event milestones with corresponding approximate time intervals.

Time Interval	Name	Description of event characteristics for time interval
[7: 00, 11: 00]	*Pre*	Before the game. Anticipated posts predicted.
[11: 00, 14: 00]	*Gates*	Gates to the venue begin opening. Anticipated posts may still be detected.
[14: 00, 14: 40]	*Entertain*	The pre-game entertainment at the venue begins.
[14: 40, 17: 00]	*Game*	The game is played. The game includes three breaks.
[17: 00, 18: 30]	*Post*	The game is finished; the audience are leaving the venue. Delayed posts are predicted.
[14: 00, 17: 00]	*Broadcast*	The live television broadcast.

Times are in Australian Eastern Standard Time.

**Table 7:** A summary of the temporal filters for datasets by content sources.

Dataset	Text	Image	Geotags
**ADon**	*[Pre,Post]*	*[Gates,Post]*	OTG: *[Gates,Game]* NOTG: *[Entertain,Game]*
**ADoff**	*[Entertain,Game]*	*[Entertain,Game]*	OTG: *[Entertain,Game]* NOTG: *[Entertain,Game]*

### Extract

Two methods are employed to extract text and image evidence from micro-blogs. Each text and image evidence is manually annotated with the category OTG or NOTG, by two researchers with experience of the case study event and research domain for cross validation of annotations. This annotated data serves two purposes:

- To provide controlled scenarios to analyze evidence testing implementations; and
- To provide the training data for supervised classification, the second method of extracting evidence.

The method for automatic geotag categorization is initially adopted from previous work [6]. Geotags were categorized as OTG if located within the MCG or a buffer surrounding the MCG. The buffer is defined by places and geographic features surrounding the MCG including train lines, roads, and other event venues.

The resulting image, text and geotag content is not manually cross-referenced per micro-blogger before combination, to enable any conflict resulting from annotation errors to be analyzed and described. For micro-bloggers with conflicting evidence, the social media accounts are inspected to verify their location during the target event, a method with precedence for social media research e.g. [12].

#### Manual annotation of text and image evidence

The ADon text content annotation process and results have been reported previously, with Cohen’s K exceeding 0.895 [6]. A new annotation experiment will apply a similar process for annotating the ADoff text content, with annotators instructed to identify the on-topic evidence types summarized in Table 3, and one additional evidence type, explicit declarations by the micro-blogger of their location at a place that is not the case study event (see examples Table 4). All other potential off-topic evidence types are left to future research. Image annotation for both ADon and ADoff have been described previously, with a Cohen’s K exceeding 0.95 reported [10]. As presented in Table 3, the image evidence is limited to that which can be categorized as on-topic.

#### Automatic text and image evidence extraction by supervised classification

The primary purpose of the supervised classification of text and image evidence in this paper is to demonstrate the possibility of an automatic method for extracting evidence and *mf* modeling from the resulting confusion matrix. Developing a state of art method for the extraction of text and image evidence is outside the scope of these experiments, with efforts from various research communities such as Natural Language Processing pursing these goals e.g. [7]. For the application domains with interest in identifying witnesses, it is suggested that the precision of the identified evidence is of importance and that false positives are minimized. For example, [57] describes the consequences of publishing false witness accounts for journalists. For these combination of reasons, attempts to utilize transfer learning to classify text and image evidence in the ADoff dataset from training models developed from the ADon dataset (similar to [10]) will not be attempted.

Previous experimentation with Weka default classifiers and feature selection filters [58] found the best results for maximizing precision of the three categories OTG, NOTG and NE in text content were achieved with a unigram model and SVM classifier [6]. However, the precision of the OTG category was less than the target 80% and recall for both the OTG and NOTG categories was less than 30% and 50% respectively. These sub-optimal results are assumed to be influenced by the lack of training samples for the OTG and NOTG categories, and the variation of evidence types for the OTG category in particular. This study attempts with new experiments to improve the classification by primarily addressing these issues. The evidence types within OTG and NOTG will be analysed to support whether classification could be improved by eliminating those with limited samples. Additionally, further training samples will be added from the Grand Final dataset.

Previous methods will again be adopted to demonstrate the automatic extraction for image evidence [10]. Preliminary evaluation of the visual bag-of-words approach to classify images in the three categories OTG, NOTG and NE found the precision for the NOTG category to be less than 45% primarily due to an inadequate sample size [59]. This study makes new experiments to introduce the NOTG category and attempts to improve classification results by adding additional training samples from the Grand Final dataset.

### Test

Three experimental scenarios are planned for evidence test implementations. The first experiments will use the manually annotated training datasets to assess the full potential to discover evidence in the off-hash datasets, and assess the results of evidence testing without the complexity of additional errors introduced by the supervised classification. The second experiment will introduce supervised classification for text and images in the on-hash dataset. This experiment demonstrates a method for computing evidence *mf* from the classifiers resulting confusion matrix and enables analysis of the impact of classification errors by comparison to the training datasets. The final experiment compares a geographically weighted approach for deriving geotag *mf*.

For the first experiments with the manually annotated training datasets, *mf* are manually assigned (see Table 8). Geotags are modeled as either OTG or NOTG, whereas a characteristic of the text and image content source is that their classification can also be NE. NE is modeled with greater certainty because the text and image datasets are unbalanced favoring this category. A belief interval of 0.1 is assigned to model uncertainty in each of these manual assignments. The *mf* for the manual annotation datasets reflects the manual annotation process does not distinguish different uncertainties for the different inference categories (*OTG* compared to *NOTG*).

**Table 8:** An example of manually assigned *mf* for evidence by content source and inference category.

Θ	*mf*					
	*G* ∩ *OTG*	*G* ∩ *NOTG*	*I* ∩ *OTG*	*I* ∩ *NOTG*	*T* ∩ *OTG*	*T* ∩ *NOTG*
{}	0	0	0	0	0	0
{*OTG*}	0.85	0.05	0.7	0.05	0.6	0.05
{*NOTG*}	0.05	0.85	0.05	0.7	0.05	0.6
{*NE*}	0	0	0.15	0.15	0.25	0.25
{*OTG*, *NOTG*}	0.1	0.1	0	0	0	0
{*OTG*, *NE*}	0	0	0	0	0	0
{*NOTG*, *NE*}	0	0	0	0	0	0
{*OTG*, *NOTG*, *NE*}	0	0	0.1	0.1	0.1	0.1

For the second experiments where text and image evidence result from a supervised classification, the predictive rate [42] computed from the confusion matrix is used to derive the corresponding *mf*. Essentially, the predictive rate *ε*_*p*_ for each class is adopted for evidence of the corresponding inference category, and 1 − *ε*_*p*_ assigned to the remainder of Θ. A *df* is then applied to introduce different inferential weights for the source of evidence relative to the other sources. A *df* = 0.6 and *df* = 0.7 are applied for evidence sourced from text and images respectively. The *mf* for geotags will remain the same as the experiments with annotated data, to simplify the assessment of supervised classification.

The final experiment explores geographically weighted methods for deriving *mf* for geotag evidence informed by [29]. Analysis of micro-bloggers who have geotag evidence NOTG conflicting with text and image evidence OTG, will support the derivation of bandwidth required by Eq (6). This approach will be compared with the decision boundary approach used in previous experiments.

The combination rule PCR6 implemented in Matlab [60] is used to compute the combination of *mf*, with order by timestamp *ts* and respecting micro-blog boundaries Eq (8) for all experiments. Ranking each micro-blogger by the *Bel(OTG)* value from the combined *mf* will be used as a tool to support analysis and comparison of results between datasets and micro-bloggers. The maximum Belief can also serve as a decision algorithm identifying the most likely witnessing status for a micro-blogger.

## Results

For all experiments, summary statistics will be reported for the ADon and ADoff datasets, or their combination labeled *ADcomb*. A summary of inference and test categories for micro-blogs and micro-bloggers by dataset are presented. Then, example micro-bloggers are selected and referred to by assigned alias for detailed analysis of the DST results.

### Training dataset experiments

Table 9 presents a summary of the case study datasets by content source at the completion of the search and filtering processes. The number of geotags and images are less by 26 and 22 respectively, compared to numbers previously reported [6], due to the addition of temporal filtering described in Tables 6 and 7.

**Table 9:** The number of content source for the ADon and ADoff datasets at the completion of the filtering processes.

	|*T*|	|*I*|	|*G*|
**ADon**	3620	245	107
**ADoff**	1224	86	85

The content source categories are text *T*, images *I*, and geotags *G*.

Table 10 presents the number of evidence categorized as *OTG* or *NOTG* by content source for the ADon and ADoff datasets. These results confirm the potential to discover a significant additional number of evidence in micro-bloggers’ off-hash datasets, which increases the number of micro-bloggers with multiple evidence for testing. The most significant *OTG* increase is 62% for geotag evidence. The greater increase in text evidence *OTG* compared with image evidence, 24% to 13% respectively, can be in part explained by the limitation to on-topic image evidence, whereas text evidence includes the new off-hash evidence type.

**Table 10:** The number of evidence by inference category and content source for the ADon and ADoff datasets.

	|*T* ∩ *OTG*|	|*T* ∩ *NOTG*|	|*I* ∩ *OTG*|	|*I* ∩ *NOTG*|	|*G* ∩ *OTG*|	|*G* ∩ *NOTG*|
**ADon**	99	129	105	23	21	86
**ADoff**	24	53	14	3	13	72
**% ADoff of ADon**^1^	24%	41%	13%	13%	62%	84%

^1^The percentage increase in the number of evidence by adding the off-hash evidence.

#### Summary evidence categorizations for micro-blogs and micro-bloggers

Table 11 present a summary of micro-blog categorization for the ADon and ADcomb datasets. The subset number of micro-blogs which intersect with *CORW* or *MIXW*, have more than one piece of evidence, that corroborates or conflicts respectively. Adding the off-hash evidence to ADon to create ADcomb, increases the number of micro-blogs categorized *OTGM* by 23%, from 184 to 227, and 42 of these contain corroborating evidence. The 53% increase in the number of micro-blogs categorized as *NOTGM*, from 221 to 338, additionally increases the number that contain corroboration, from 15 to 25. Three of the total 70 micro-blogs with more than one piece of evidence are categorized *MIXW* indicating conflict.

**Table 11:** The number of micro-blogs by summary inference category *CM* and corroboration for the ADon and ADcomb datasets.

	|*OTGM*|	|*OTGM* ∩ *CORW*|	|*NOTGM*|	|*NOTGM* ∩ *CORW*|	|*MIXW*|
**ADon**	184	37	221	15	2
**ADcomb**	227	42	338	25	3
**% change ADon to ADcomb**^1^	23%	14%	53%	67%	50%

^1^The percentage increase in the number of micro-blogs for each category by the addition of the off-hash evidence.

Table 12 presents a summary of micro-blogger categorization for the ADon and ADcomb datasets. The subset number of micro-bloggers that intersect *COR* or *MIXB* have more than one piece of evidence, that corroborates or conflicts respectively. It is interesting that the addition of the off-hash data increased the number of micro-bloggers with conflict, in particular seven micro-bloggers change categorization from *OTGB* to *MIXB*. As expected the number of micro-bloggers with corroboration increased, but in greater numbers for those categorized *NOTGB* by 110%.

**Table 12:** The number of micro-bloggers by summary inference category *CB* and corroboration for datasets ADon and ADcomb.

	|*OTGB*|	|*OTGB* ∩ *COR*|	|*NOTGB*|	|*NOTGB* ∩ *COR*|	|*MIXB*|
**ADon**	146	52	171	29	5
**ADcomb**	139	62	170	61	13
**% change ADon to ADcomb**^1^	-5%	19%	-1%	110%	160%

^1^The percentage change in the number of micro-bloggers for each category by the addition of the off-hash evidence.

#### DST evidence combination for case study micro-bloggers

Table 13 presents DST combination and summary categorization results for eight selected micro-bloggers. The *Bel(OTG)* values extracted from the combined *mf* appear to reflect where multiple evidence is present for a micro-blogger, and whether these evidence conflict or corroborate. For example, five micro-bloggers have a categorization of *OTGB* for the ADon dataset, and four of these with corroboration have *Bel(OTG)* values greater than 0.95. Sensor321 with a *Bel(OTG)* less than 0.01 communicates multiple evidence corroborating NOTG. The *Bel(OTG)* value of 0.311 for Sensor150 and Sensor151, appear to reflect the uncertainty of the *MIXB* categorization.

**Table 13:** The summary inference category *CB*, corroboration *COR*, and *Bel(OTG)* value and corresponding rank, for the example micro-bloggers by the ADon and ADcomb datasets.

	ADon				ADcomb			
Alias	*CB*	∈ *COR*	*Bel(OTG)*^1^	rank^2^	*CB*	∈ *COR*	*Bel(OTG)*^1^	rank^2^
**Sensor1**	*OTGB*	✓	0.996	1	*OTGB*	✓	0.996	1
**Sensor6**	*OTGB*	✓	0.981	5	*MIXB*	-	0.984	10
**Sensor8**	*OTGB*	✓	0.953	10	*OTGB*	✓	0.986	6
**Sensor14**	*OTGB*	✓	0.955	9	*OTGB*	✓	0.986	7
**Sensor129**	*OTGB*	-	0.6^3^	20	*MIXB*	-	0.439	31
**Sensor150**	*MIXB*	-	0.311	22	*MIXB*	-	0.001	60
**Sensor151**	*MIXB*	-	0.311	22	*MIXB*	-	0.574	29
**Sensor321**	*NOTGB*	✓	0.003	33	*NOTGB*	✓	0.003	56

^1^The *Bel(OTG)* values are rounded to three decimal places for presentation purposes.

^2^The rank of the case study micro-blogger computed from the *Bel(OTG)* value relative to the set of micro-bloggers with evidence for the dataset.

^3^Micro-bloggers with a single piece of evidence and therefore not subject to evidence combination.

**Fig 10:**
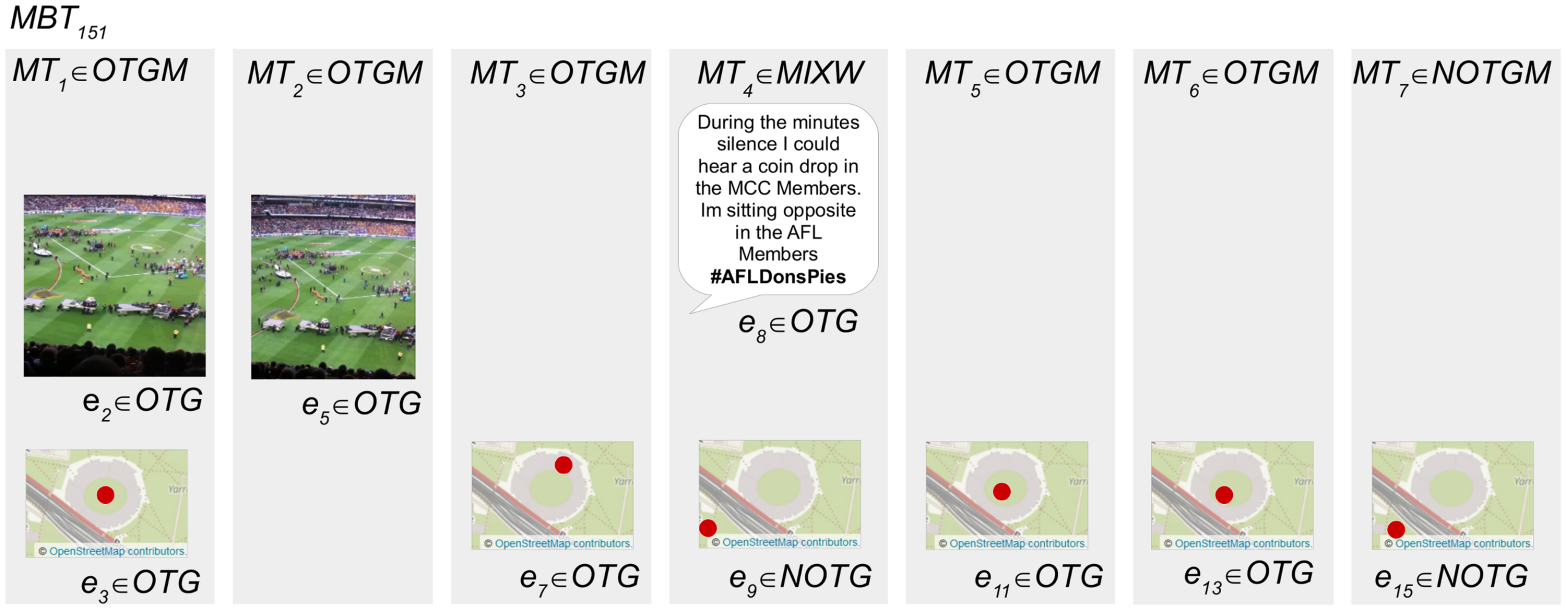
Micro-blogs and evidence identified for Sensor151. The conflicting evidence *e*_15_ is identified at the end of the timeline. The image and text content are similar to that posted by the micro-blogger from the case study, and are for illustrative purposes. The images in the figure are printed under a CC BY license, with permission from Rachael Hopkins, original copyright 2017.

**Fig 11:**
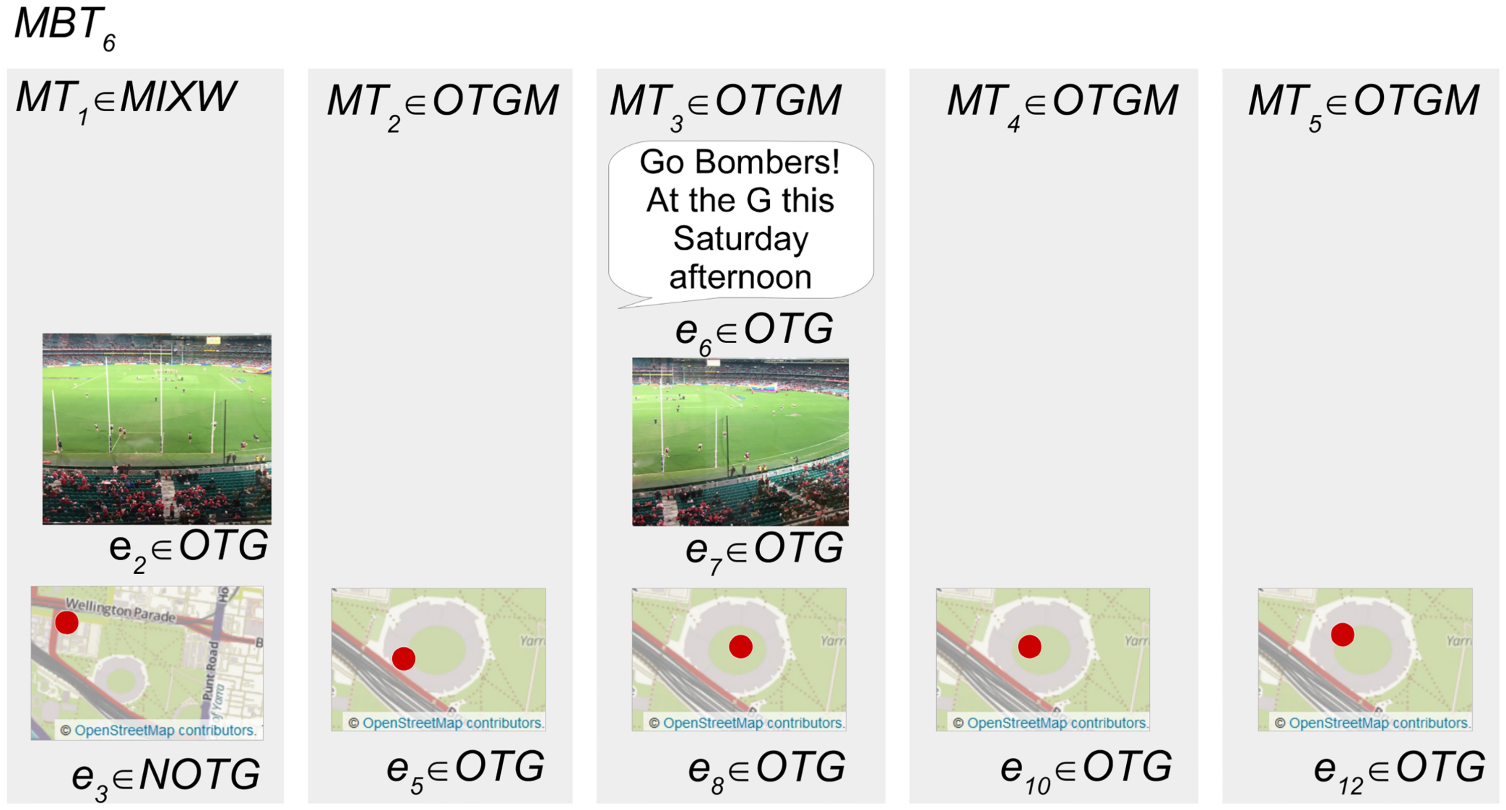
Micro-blogs and evidence identified for Sensor6. The conflicting evidence *e*_3_ is identified at the beginning of the timeline. The image and text content are similar to that posted by the micro-blogger from the case study, and are for illustrative purposes. The images in the figure are printed under a CC BY license, with permission from Rachael Hopkins, original copyright 2017.

Interestingly for the ADcomb dataset, the *Bel(OTG)* values for micro-bloggers with mixed inference categorization *MIXB* support two interpretations. Sensor6 and Sensor150 both have conflicting evidence, but *Bel(OTG)* values of 0.984 and 0.001 strongly support a status of OTG and NOTG respectively, despite this conflict. Whereas, the *Bel(OTG)* values of 0.439 and 0.574 for Sensor129 and Sensor151 respectively, communicate that the conflict is significant. The *OTG* belief interval for Sensor129 is in fact borderline [0.439,0.539]. To support further analysis, the evidence extracted for Sensor151 and Sensor6 are presented in Figs 10 and 11 respectively. For Sensor 151, the final evidence to be combined is a conflicting geotag, *e*_15_, which has a greater influence than all the preceding evidence. Comparatively, the conflicting geotag *e*_3_ for Sensor6 occurs at the beginning of the timeline. This may be an advantage for some real-time applications, an alternative view is that the result does not summarize the majority *OTG* evidence. It is not possible to interpret directly from the *Bel(OTG)* value or *mf* the number of evidence or what content source it was extracted from. Such a metric for summarizing evidence is identified for future work.

One purpose of the ranks derived from the *Bel(OTG)* values in Table 13 are to assess the number of different evidence combinations in each dataset. Sensor321, with the lowest rank of 33 in ADon dataset provides the number of different evidence combinations that can be derived by the *Bel(OTG)* value. The number of ranks increases to 56 for the ADcomb dataset due to the addition of evidence for the same number of micro-bloggers. As predicted in the Theory Section, evidence combinations that cannot be distinguished by *mf* combination results were identified, and as such are not differentiated in the ranks. For example, the structure of evidence differs for Sensor67 and Sensor15 presented in Eqs (11) and (12) respectively, both have corroboration but Sensor67 is within a single micro-blog whereas Sensor15 is between micro-blogs. Although this difference can be identified from their set representations it cannot be identified from the combined *mf*. A metric that can additionally differentiate this structure is identified for future work.

$$\begin{array}{c} MBT_{67}=\left\{MT_{1}=\left\{e_{1},e_{2}\right\}\right\}\text{,}\;\text{where}\;e_{1}\in G\cap OTG\text{,}\;e_{2}\in I\cap OTG \end{array}$$ (11)

$$\begin{array}{c} MBT_{15}=\left\{MT_{2}=\left\{e_{3}\right\}\;\text{,}MT_{3}=\left\{e_{4}\right\}\right\}\text{,}\;\text{where}\;e_{3}\in G\cap OTG\text{,}\;e_{4}\in I\cap OTG \end{array}$$ (12)

#### Conflict in the training datasets

Conflict was expected in the training datasets for a number of reasons. Although the inter-annotator agreement for text and image evidence was strong it was not complete, and previous experiments suggested the possibility of inaccurate GPS. Manual inspection verified that the three micro-blogs identified with *MIXW* categorization in the ADcomb dataset (Table 11) reveals two scenarios causing conflict. The first scenario is caused by geotags categorized *NOTG* posted with text or images *OTG*, for example *MT*_4_ in Fig 10 and *MT*_1_ in Fig 11. As these micro-blogs were posted during the event it suggests the decision boundary drawn for geotag categorization rather than inadequate temporal filtering, is the cause. The second scenario is that text evidence is incorrectly labeled by multiple annotators, and examples are provided in Table 14. With inspection of each corresponding micro-blogger complete posting history, it is apparent the location context of these text evidence are genuinely difficult to interpret due to the event being broadcast live and differing interpretations of place boundaries. Analysis of the 13 micro-bloggers identified in the ADcomb dataset with categorization *MIXB* (Table 12) confirms the same two scenarios are causing conflict between micro-blogs. It is concluded therefore, that the cause of conflict within micro-blogs or between micro-blogs, does not differ for this case study.

**Table 14:** Example miss-classified text by human annotators.

Example text
a) *The crowd is starting to build! @MCG #ANZACDay2015 #AFLDonsPies #gopies* [61]
b) *The @MCG, from any vantage point, is simply MAGNIFICENT. We’re so lucky to have it here in #Melbourne. #AFLDonsPies* [62]
c) *Hey @Optus I’d love to enter the @mcg crowd comp but i think i need reception for that! I’m not in a cave, I’m in the CBD*… [63]

a) and b) were incorrectly annotated OTG, and c) incorrectly annotated NOTG because the place MCG was not considered to be within the Central Business District (CBD) by the annotators.

### Experiments with evidence extracted by supervised classification

The text and image classification results presented in Table 15 achieve the targets set for this research, an improvement on precision for all classes from previous experiments [6]. For the text evidence, 57 samples from the *OTG* and *NOTG* classes were reclassified as *NE* for the ADon dataset. *OTG* samples were restricted to the evidence type indicating explicit location context at the event, and 47 samples of similar evidence type were added from the Grand Final datasets to improve the training model. These efforts improved the homogeneity of evidence types representing the *OTG* category. As with previous experiments, the best classification was achieved with Weka’s SMO classifier [58]. However, in addition to unigrams [6], bigrams, trigrams, and parts-of-speech features, were created before Weka’s attribute selection filter applied. Although the recall are still sub-optimal, the enhancements described improved the precision of the evidence classified as *OTG* and *NOTG* with both exceeding 80%. For the image classification, the *NOTG* class was included, 12 non-typical *OTG* images were reclassified as *NE*, and an additional 53 *OTG* and 70 *NOTG* image samples were added from the Grand Final dataset.

**Table 15:** A summary of text and image classification results.

	Text		Image	
Class	Precision	Recall	Precision	Recall
**OTG**	0.803	0.588	0.978	0.943
**NOTG**	0.852	0.730	0.854	0.911
**NE**	0.913	0.965	0.912	0.912

#### Summary supervised classification dataset results

The datasets resulting from supervised classification are labeled *ADon_a* and *ADcomb_a*. The number of micro-blogs with evidence in the ADon_a dataset is reduced to 312 from the 407 identified in the training dataset ADon. This reduction is primarily due to the reclassification of non-typical samples and the sub-optimal recall for text evidence. Additionally, the number of micro-blogs with evidence in the ADcomb_a dataset is reduced due to the inclusion of geotag evidence only. Transfer Learning for text and images were not attempted. Table 16 presents the number of micro-blogs by combined inference category, and the number of these that change combined inference category from the training datasets ADon and ADcomb. For example, 132 micro-blogs in ADcomb_a were categorized *OTGM*, and six of these micro-blogs were categorized with a different inference categorization (either *NOTGM* or *MIXW*) in the training dataset. These results communicate, that in addition to a reduction in the number of evidence, the impact of supervised classification can be to change a micro-blogs inference categorization.

**Table 16:** The number of micro-blogs by combined inference category *CM* and corroboration for the ADon_a and ADcomb_a datasets.

	|*OTGM*|	|*OTGM* ∩ *CORW*|	|*NOTGM*|	|*NOTGM* ∩ *CORW*|	|*MIXW*|
**ADon_a**	124	23	183	7	5
**Change from ADon**^1^	6	0	23	0	5
**ADcomb_a**	132	23	253	7	5
**Change from ADcomb**^2^	6	0	24	0	5

^1^The subset number of micro-blogs in the ADon_a dataset that changed categorization from the training dataset ADon.

^2^The subset number of micro-blogs in the ADcomb_a dataset that changed categorization from the training dataset ADcomb.

The number of micro-bloggers with evidence in the ADon_a dataset is reduced to 240, from 322 in the training datasets, including five who were not previously identified with evidence. Table 17 presents the number of micro-bloggers for each summary inference category, and the number of these that change category from the training datasets ADon and ADcomb. For example, 96 micro-bloggers in ADcomb_a were categorized *OTGB*, and five of these micro-bloggers were categorized with a different inference categorization in the training dataset. Unlike the micro-blogs, a single micro-blogger Sensor207 changed categorization to *OTGB* with corroboration, meaning two evidence where automatically classified for this micro-blogger *OTG* where no evidence were categorized *OTG* in the training dataset.

**Table 17:** The number of micro-bloggers by summary inference category *CB* and corroboration category for the ADon_a and ADcomb_a datasets.

	|*OTGB*|	|*OTGB* ∩ *COR*|	|*NOTGB*|	|*NOTGB* ∩ *COR*|	|*MIXB*|
**ADon_a**	98	30	132	16	10
**Change from ADon**^1^	4	1	14	0	9
**ADcomb_a**	96	29	131	31	13
**Change from ADcomb**^2^	5	1	13	1	6

^1^The subset number of micro-bloggers in the ADon_a dataset that changed categorization from the training dataset ADon.

^2^The subset number of micro-bloggers in the ADcomb_a dataset that changed categorization from the training dataset ADcomb.

#### DST combination of evidence extracted by supervised classification

Table 18 presents the *Bel(OTG)* value from the combined *mf* result for the example micro-bloggers. The source *mf* are derived from the predictive rate, computed from the supervised classification results in Table 15. Analysis of the ADon_a dataset results show with the exception of Sensor6, all micro-bloggers have less evidence detected, which has changed three micro-bloggers’ summary inference category. Sensor129 no longer has evidence detected, vulnerable to sub-optimal recall of text evidence. Sensor207 has a *Bel(OTG)* value of 0.73 for two text evidence *OTG*, a number less than micro-bloggers with evidence extracted from a variety of sources.

**Table 18:** The summary inference category *CB*, corroboration *COR*, and *Bel(OTG)* value and corresponding rank, for example micro-bloggers by the ADon_a and ADcomb_a datasets.

	ADon_a				ADcomb_a			
Alias	*CB*	∈ *COR*	*Bel(OTG)*^1^	rank^2^	*CB*	∈ *COR*	*Bel(OTG)*^1^	rank^2^
**Sensor1**	*OTGB*	✓	0.991	1	*OTGB*	✓	0.991	1
**Sensor6**	*OTGB*	✓	0.981	2	*MIXB*	-	0.972	5
**Sensor8**	*MIXB*	-	0.723	15	*MIXB*	-	0.983	3
**Sensor14**	*OTGB*	✓	0.955	6	*OTGB*	✓	0.985	2
**Sensor129**	-	-	-	-	-	-	-	-
**Sensor150**	*NOTGB*	-	0.05^3^	23	*NOTGB*	✓	0.003	41
**Sensor151**	*NOTGB*	-	0.05^3^	23	*MIXB*	-	0.414	21
**Sensor321**	*MIXB*	-	0.003	34	*MIXB*	-	0.003	42
**Sensor207**	*OTGB*	✓	0.730	13	*OTGB*	✓	0.730	15

^1^The *Bel(OTG)* values are rounded to three decimal places for presentation purposes.

^2^The rank of the case study micro-blogger computed from the *Bel(OTG)* value relative to the set of micro-bloggers with evidence for the dataset

^3^Micro-bloggers with a single piece of evidence and therefore not subject to evidence combination.

Excluding Sensor129 and Sensor207, it appears the inclusion of the off-hash geotag evidence from the ADcomb_a dataset confirms the micro-bloggers’ status, and is consistent with the training dataset ADcomb. The results are robust to errors introduced from the supervised classification for the example micro-bloggers. The changes in summary inference category *CB* might serve to alert that a conflict is detected in the chain of evidence, which can be further investigated if required. No evidence was discovered for Sensor207 off-hash, to corroborate or conflict this micro-blogger’s status, which results in a recommendation for future research. When conflict is not detected, the status of a micro-blogger might still be questioned if the evidence is limited to a single source/ evidence type.

Previously, it has been explained that the number of ranks can reflect the number of different combinations of evidence to be found in that dataset. However, the total number of ranks for the ADon_a dataset in Table 18 are similar to those for the ADon dataset, 34 compared to 33, even though the number of evidence and micro-bloggers are significantly less. This suggests that the automated classification introduced not just classification errors, but increased the different combinations of evidence, that introduces the possibility that micro-bloggers post evidence in patterns. This identifies an avenue of future research.

### Geotags

**Fig 12:**
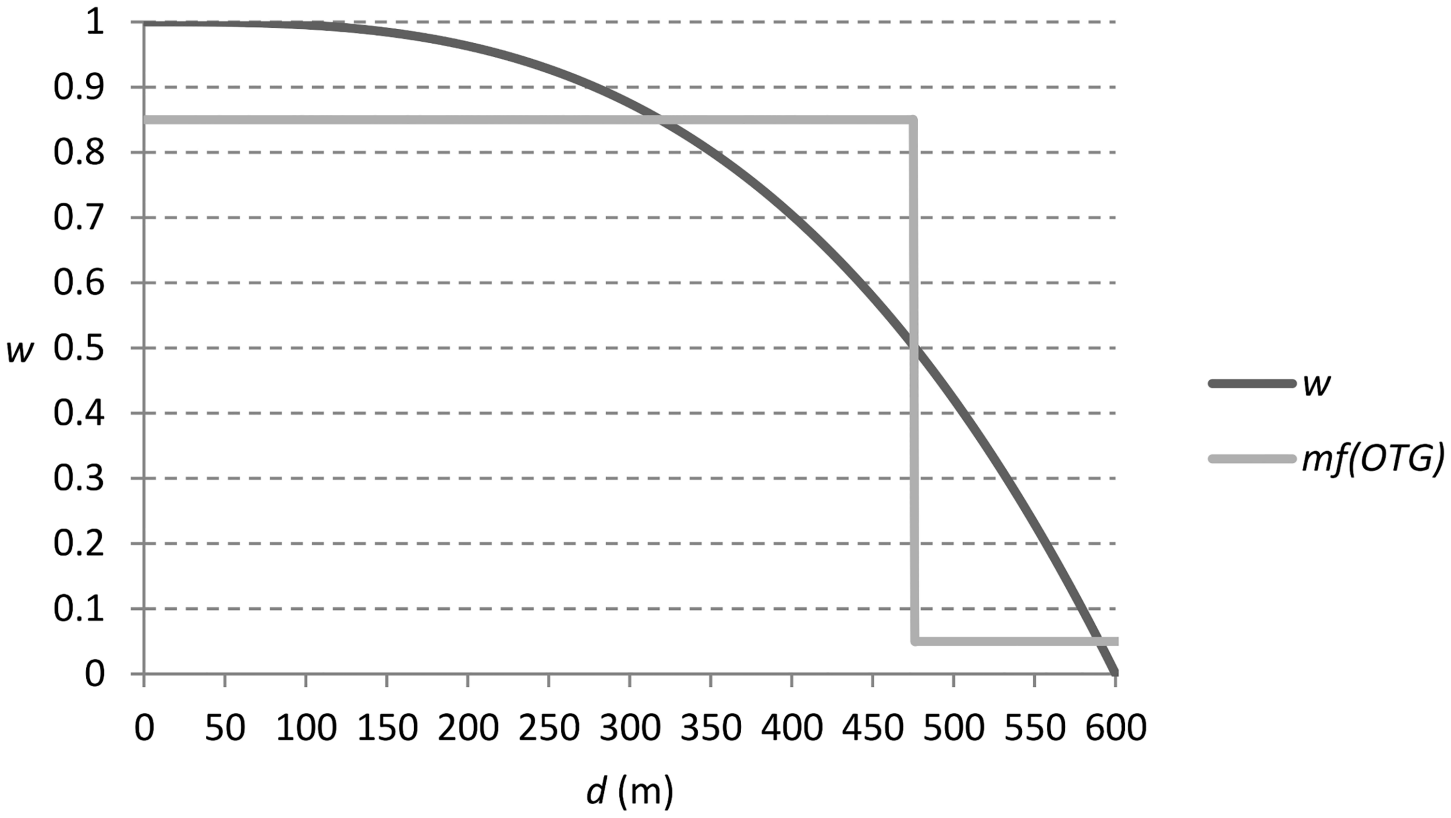
A comparison of approaches for deriving *mf* for geotag evidence. *w* is computed using a Tri-cube Kernel function Eq (6), and *mf(OTG)* is a manual assignment based on a decision boundary for categorization of OTG or NOTG.

This section provides results of experiments exploring a geographically weighted approach to deriving *mf* for geotag evidence, which can be summarized by Fig 12. The *mf(OTG)* curve in Fig 12 is the approach where first a spatial decision boundary is established from the events geographic extents, followed by a categorization of evidence as *OTG* or *NOTG*, and finally manual assignment of *mf* by experts. Previous analysis of conflict suggests extension of the decision boundary for this case study to approximately 475m from the center of the MCG. The *w* curve in Fig 12 presents the results of Eq (6) with bandwidth *b* = 600 for varying distances *d* from the center of the event venue. In [29] the *w* value is adopted to configure *mf*. The value for *b* was established though experimentation as the approximate value where *w* at *d* = 475m switches from greater certainty OTG to NOTG, that is *w* = 0.5. Fundamentally, in this geographically weighted approach the categorization of OTG or NOTG is derived by the computed *w*.

The differences resulting from the geographically weighted approach are apparent from Fig 12. However, a primary question is although a geotag may be located within an event venue, expert knowledge indicates it should not be assigned an absolute certainty value *OTG*, both to reflect the possibility the location is in error due to limitations of the source sensors (e.g. GPS), and to be able to distinguish results where multiple evidence corroborates an *OTG* hypothesis. Further questions include what model to adopt for geotag evidence where *d* > *b*, and the method for assigning (1 − *w*) to Θ. The results of this experiment identify the potential to incorporate a geographically weighted approach, but recommend further research is required before implementation can proceed.

## Discussion

The results indicate the evidence model implemented to test and combine evidence for the test process of the framework (see Fig 1), was effective for categorizing the witnessing status of micro-blogs and micro-bloggers for the case study event. For example, 19% of the 227 micro-blogs categorized with evidence *OTG* could be categorized as having multiple evidence with corroboration. And significantly for testing purposes, 45% of micro-bloggers categorized with evidence *OTG* could be further categorized as having corroboration, confirming the benefit of evidence combination by micro-blogger in comparison to individual micro-blogs. Additionally, the modeling of the counter-evidence category *NOTG* enabled the distinction between conflicting evidence within a single micro-blog, or between micro-blogs for a micro-blogger. In the training dataset, thirteen of the 136 micro-bloggers with multiple evidence were identified to have conflicting evidence. A detailed analysis of this conflict identified it was caused by two sources of measurement error, geotags categorized *NOTG* because they were outside the event venue, and incorrectly assigned categories to text content by annotators. The results indicate more restrictive temporal filtering of geotag evidence (Table 7) resolved conflict identified in previous research [6], due to varying temporal characteristics in comparison to text.

Additionally the results indicate the potential benefits of incorporating off-hash datasets discovered by the search micro-blogger processes of the framework (see Fig 1), with increased numbers for all inference categories and evidence types detected. The largest potential increase was 84% for geotags *NOTG*, an expected finding as all geotags can be considered evidence in comparison to text and images, which are limited to on-topic evidence types (see Table 3). Nevertheless, the potential increase in *OTG* evidence identified in the training datasets for text and images, is 24% and 13% respectively (see Table 10).

The new supervised classification experiments for automatic extraction of text and image evidence, improved the precision of results to exceed the target 80% for all classes, in comparison to previous research [6, 10]. The updates included introducing further training samples for the *OTG* and *NOTG* classes for both the text and image experiments from a similar event instance, and pruning atypical samples to achieve a more homogeneous representation of the evidence types adopted for each class. However, the recall for text evidence was still sub-optimal, and one reason the supervised classification identified evidence for 240 micro-bloggers, a reduction from the potential 322 identified with the training datasets. Another reason is this research did not pursue transfer learning to discover on-topic text and image evidence in the off-hash datasets, a trade-off to maintain the precision of extracted evidence. However, the results are still an increased number of micro-bloggers distinguished from baseline methods that focus on geotags alone. Classification errors resulted in six micro-bloggers being incorrectly allocated evidence *OTG* including one with corroboration.

The evidence model also supported a DST implementation for evidence combination testing the witnessing status of individual micro-bloggers. The DST evidence combination results indicated that a conflicting piece of evidence in a micro-blogger’s posting timeline, may have a negligible impact on the micro-blogger’s combined *mf*, or dominate the results. Analysis revealed this is because the last evidence posted had greater influence than previous evidence combinations, a potential advantage for real-time applications. Additionally, similar DST combination results for the training and supervised classification datasets (Table 13 compared with Table 18), indicate a robustness to a reduction in evidence and classification errors, compared to the inference categorization (Table 17). However, during analysis of this behavior it was noted that the *mf* combination result offered limited transparency as to the contributing evidence. For example, it was not possible to assess a *balanced of evidence*, a comparison of the number of evidence posted by the micro-blogger categorized *OTG* versus *NOTG*.

In the training dataset experiments, the relative uncertainty of evidence was simply distinguished by content source, and represented by a manually configured *mf*. With the supervised classification of text and image evidence, the varying uncertainty for each inference category represented by the classifiers confusion matrix, was incorporated into the *mf*. In this scenario, although multiple uncertainties were being modeled for evidence derived from the same content source, the evidence was still bound by the inference categorization of *OTG* and *NOTG*. Exploration of a geographic weighting approach for the derivation of a *mf* for geocode evidence, introduced a method for deriving uncertainty that is not primarily from the evidence (or content) type or inference category. However, it was concluded that a number of questions require further research before adoption is recommended. For example, although a geotag may be located within an event venue, an absolute certainty value may not be ideal, both to reflect the possibility the location is in error and to be able to distinguish where multiple evidence corroborates an *OTG* hypothesis.

## Conclusions and future work

The primary contributions of this research have been to demonstrate a complete framework of processes for identifying potential witnesses of a case study event, with particular focus for this paper on evidence testing. A generalized evidence model has been defined that has supported a DST test implementation. Additionally, it is confirmed that the number of evidence available for this test are increased by searching a micro-blogger’s off-hash posts during the event. It was also confirmed that the proportion of evidence that could be tested was significantly increased by combining evidence for a micro-blogger in comparison to individual micro-blogs. These contributions are significant as witnesses posting from on-the-ground *OTG* are typically a small fraction of micro-bloggers posting about an event e.g. [12], and concerns for miss-leading information in social media.

The evidence model has defined sets to support the categorization of evidence, micro-blogs, and micro-bloggers, with regards to inference categories, and corroboration and conflict. The model was successfully applied to a case study event, enabling summaries of micro-bloggers’ status to be presented. These summaries confirm the potential benefits of incorporating off-hash datasets, with increased numbers for all categories of evidence detected, the greatest being 84% for geotags not on-the-ground *NOTG*.

Evidence was defined as being extracted from the text, image, or geotag content of micro-blogs, and characterized by varying uncertainty, which are modeled via mass functions *mf* in a DST implementation. A range of experiments were completed to explore the development of *mf* including manual configuration by experts for training datasets, and derivation from a classifiers confusion matrix to demonstrate automatically extracted datasets. The results indicated particular DST behaviors that include conflict might have significant or negligible impact, depending on the order and number of evidence combined, however, the results offered limited transparency as to the contributing evidence.

The methods selected to derive *mf*, and combination algorithm, adequately responded to individual micro-bloggers’ scenarios for this case study. However, in response to the identified complexity and limited transparency of results, the development of a metric to provide a *balance of evidence* is proposed for future research. This metric could include a count of evidence for each micro-blogger, with each evidence type weighted so the result provides transparency on that included. Additionally, it could support analysis of whether conflict is significant in comparison to the total count of evidence for the micro-blogger, include factors that distinguish between structural differences in evidence posting, and identify if corroboration is from a variety of evidence types. It is also intended that additional future work can explore alternative formal approaches for evidence combination such as Fuzzy Sets and Possibility theory, supported by the evidence models described in this study.

For text and image evidence varying uncertainties for different evidence types are envisaged in future implementations. There are currently limited accessible methods to automatically extract the evidence types that have been identified with manual annotation. State of the art machine learning indicates that all evidence will not be extracted with a single method, it is likely that numerous highly specialized methods will be employed for specific evidence types. For example, the needs of this research are methods for extracting spatial and temporal context from text content. In such a scenario, a requirement of the test processes will be handling varying inferential weight for different evidence types, and varying uncertainties that would be associated with different extraction methods for these evidence types. These new evidence extraction methodologies can be tested and utilized by the framework in future work.
